# Phthalocyanine-Functionalized Magnetic Silica Nanoparticles as Anion Chemosensors

**DOI:** 10.3390/s21051632

**Published:** 2021-02-26

**Authors:** João M. M. Rodrigues, Andreia S. F. Farinha, Zhi Lin, José A. S. Cavaleiro, Augusto C. Tome, Joao P. C. Tome

**Affiliations:** 1 LAQV-REQUIMTE, Department of Chemistry, University of Aveiro, 3810-193 Aveiro, Portugal; jcavaleiro@ua.pt (J.A.S.C.); actome@ua.pt (A.C.T.); 2 Water Desalination and Reuse Center (WDRC), Division of Biological and Environmental Sciences (BESE), King Abdullah University of Science and Technology (KAUST), Thuwal 23955-6900, Saudi Arabia; 3 CICECO and Department of Chemistry, University of Aveiro, 3810-193 Aveiro, Portugal; zlin@ua.pt; 4 CQE, Departamento de Engenharia Química, Instituto Superior Técnico, Universidade de Lisboa, 1049-001 Lisboa, Portugal

**Keywords:** anions, anion binding, chemosensor, magnetic nanoparticles, phthalocyanines

## Abstract

Anionic species are one of the most common pollutants in residual and freshwaters. The presence of anthropogenic anions in water drastically increases the toxicity to living beings. Here, we report the preparation of a new optical active material based on tri(tosylamino)phthalocyanines grafted to ferromagnetic silica nanoparticles for anion detection and removal. The new unsymmetrical phthalocyanines (Pcs) proved to be excellent chemosensors for several anions (AcO−, Br−, Cl−, CN−, F−, H_2_PO_4_−, HSO_4_−, NO_2_−, NO_3_−, and OH−) in dimethyl sulfoxide (DMSO). Furthermore, the Pcs were grafted onto magnetic nanoparticles. The resulting novel hybrid material showed selectivity and sensitivity towards CN−, F−, and OH− anions in DMSO with limit of detection (LoD) of ≈4.0 µM. In water, the new hybrid chemosensor demonstrated selectivity and sensitivity for CN− and OH− anions with LoD of ≈0.2 µM. The new hybrids are easily recovered using a magnet, allowing recyclability and reusability, after acidic treatment, without losing the sensing proprieties.

## 1. Introduction

The development of new technologies for molecular recognition based on nanoparticles has received massive attention from the scientific community [1,2,3,4]. Nevertheless, the preparation of selective chemosensors, especially for detection, quantification, and/or removal of anions from polluted waters, is still challenging [4,5,6,7,8,9,10]. The recognition of a specific anion by a receptor can induce a response, being it an electrochemical or optical transition (e.g., color change or fluorescence ON/OFF), that can be used to monitor the presence and, in some cases, the concentration of an anionic guest [11,12]. This performance is predominantly useful for the detection of environmentally harmful anions (e.g., nitrate and phosphate) [13,14], detection of potentially toxic anions (e.g., cyanide) [15], or in medical diagnostics [16]. Therefore, the preparation of nanomaterials with optical active chemosensors units (chromogenic and/or fluorogenic) can provide an easy and quick assessment of those anions [17,18].

In particular, silica nanoparticles (SNPs) have been intensively studied in many scientific and technological areas due to their chemical and physical properties [19]. In the biomedical field, these materials and their hybrids have been studied, not only as “smart” systems for drug delivery but also for enzyme immobilization [20,21,22,23,24]. In this field, magnetic silica nanoparticles (MSNPs), which present the SNP characteristics in conjugation with a magnetic core, have been receiving significant attention. The magnetic core of such materials increases their applications since they can be easily driven, removed, or recycled using a magnetic field [25,26,27,28,29,30]. These properties boost their development and use in several research fields, e.g., engineering or materials science, environmental, biotechnology and biomedical areas [31,32,33]. We previously demonstrated the possibility of coupling different tetrapyrrolic macrocycle dyes to MSNPs for the photodynamic inactivation of microorganisms and the photodegradation of medicinal drugs [29,30,34,35].

The detection of anions in solution through a solid material can only happen if the anion is relocated from the (aqueous) solution to the solid phase. The success of the process can be accomplished by following three different approaches: (1) by anion receptors in the solid state high affinity to anions; (2) by grafting anion receptors onto membranes or solid materials; and (3) by adding organic anion receptors to aqueous solution inducing precipitation or crystallization of the anion−receptor complex [36]. One of the main advantages of using solid-phase materials is that it is generally easier to separate the liquid-donating phase from the receiving solid phase, turning these approaches into a cost-effective technique [36]. Recent reports describe the use of Merrifield resin (MR) as solid state chemosensor for the detection of anionic species [37]. The MR was firstly functionalized with specific probes (derived from Brooker’s merocyanine), and then evaluated as chemosensor for the detection of anionic species. The experiments were made in trichloromethane solvent and the results show a fluorescence change in the presence of CH_3_COO−, F−, H_2_PO_4_−, and CN− anions. The MR chemosensor were also demonstrated to hold chromogenic properties, due to the coupling of the probes, allowing color change when in the presence of the previous described anions [37]. The versatility of using MR as chemosensor is related with the fact that the MR is insoluble in water and organic solvents. This property allows the interaction with anions to be performed in the solid phase with the easy separation and purification of reagents, intermediates, and reaction products.

Benzimidazoles were also reported as solid state chemosensors for anions [38]. 5,6-Dimethyl-2,2′-bis-1*H*-benzimidazole was shown to possess high sensitivity and selectivity for F− and AcO− anions in solid state as well as in acetonitrile medium. Through the interaction of the anion with the amine groups of the chemosensor, the –NH deprotonation mechanism occurs, thus promoting a distinct color change, via naked eye detection, from yellow to deep green and then to brown [38].

In this context, the use MSNPs as chemosensors, especially for water analysis, demands that they meet some important requirements, such as: (i) efficient interaction with the target analytes in aqueous media, even at very low concentrations; (ii) to be recovered with minimum loss; and (iii) to be reusable.

In 2014, we showed, for the first time, the ability of phthalocyanines (Pcs) to be used as efficient chromogenic chemosensors for anions [39]. Later, we reported that a Pc containing four tosylamino groups (and different metal ions in the macrocycle core) may be used as selective chemosensors for cyanide in aqueous solutions [40]. Herein, we describe the development of asymmetric zinc Pcs bearing three tosylamino groups (**Pc1**) and their immobilization on MSNPs (**MSNP-Pc1**). The detection and quantification of anions with **Pc1** was made in DMSO as **Pc1** is not water soluble.

To the best of our knowledge, this is the first time that magnetic nanoparticles functionalized with Pcs are used as anion chemosensors. The new **Pc1** incorporates a tetrafluoroisoindole unit that allowed the hybridization. The anion binding studies were done in DMSO for **Pc1**, and in DMSO and water for **MSNP-Pc1**.

## 2. Methods and Materials

### 2.1. Reagents

All reagents and solvents were used as received from commercial sources without further purification. Tetrabutylammonium (TBA) salts of acetate, bromide, chloride, cyanide, fluoride, dihydrogen phosphate, hydrogen sulfate, nitrate, nitrite, and hydroxide, all purchased from Sigma-Aldrich, were used as the anion sources.

### 2.2. Apparatus

^1^H, ^19^F, and ^13^C NMR spectra were recorded on a Bruker Avance-300 spectrometer at 300.13, 282.40, and 75.47 MHz, respectively, using tetramethylsilane as internal reference. The HRMS spectra were recorded on a LTQ Orbitrap XL mass spectrometer using 3-nitrobenzyl alcohol (NBA) as matrix. For anion binding inspection, the absorption and emission spectra were recorded on a Shimadzu UV-2501-PC spectrophotometer and on a Fluorolog Tau-3 spectrofluorometer, respectively.

### 2.3. Synthesis

#### 2.3.1. 1,2,3,4-Tetrafluoro-9(10),16(17),23(24)-tri(tosylamino)phthalocyaninatozinc(II) (Pc1)

In a sealed tube, 4-tosylaminophthalonitrile (500 mg, 1.68 mmol), tetrafluorophthalonitrile (57.0 mg, 0.28 mmol), and zinc acetate (93.5 mg, 0.64 mmol) were dissolved in 1-chloronaphthalene (5 mL). The reaction mixture was allowed to react at 150 °C for 24 h. The resulting green mixture was cooled down to room temperature, and water was added to induce the precipitation of the Pcs. After filtration, the solid was washed with water and then dissolved in dichloromethane/methanol (1:4). The organic phase was then concentrated, re-dissolved in tetrahydrofuran (THF), and purified by flash column chromatography, using a gradient of THF/hexane (1:1 to 3:1) as eluent. The desired tri(tosylamino) phthalocyanine **Pc1** (a mixture of isomers) was the fourth fraction coming out and was precipitated from dichloromethane/hexane (36 mg, 6% yield). **^1^H NMR** (300.13 MHz, DMSO-d*_6_*): δ 2.26 (s, 9H, CH_3_), 7.36–7.46 (m, 6H, *o*-Ts H), 7.91–8.04 (m, 9H, *m*-Ts H and *β*-H), 8.91–9.08 (m, 6H, *α*-H), 11.17 (s, 3H, NH). **^19^F NMR** (282.40 MHz, DMSO-d*_6_*): δ −177.84 to −174.50 (m, 2F, *β*-F), −165.98 to −163.06 (m, 2F, *α*-F). **^13^C NMR** (75.47 MHz, DMSO-d*_6_*): δ 21.0 (CH_3_), 27.4, 30.8, 25.8, 45.7, 58.0, 67.0, 69.3, 112.5, 121.5, 123.5, 127.0, 130.0, 133.4, 136.8, 139.0, 139.7, 143.6, 152.6, 162.3. **UV–Vis** (DMSO) λ_max_ (log *ε*): 355 (4.1), 680 (4.9) nm. **UV–Vis** (solid-state) λ_max_: 400, 715 nm. **HRMS** (ESI) *m*/*z*: calculated for C_53_H_37_F_4_N_11_O_6_S_3_Zn [M+4H]^+^: 1159.9005; found: 1159.9999.

Preparation of magnetic silica nanoparticles (**MSNP**)

The preparation of silica coated magnetite nano-support is very similar to the one in the literature [29]. The Fe_3_O_4_ core was obtained by the conventional co-precipitation method. First, 2.95 g of FeNH_4_(SO_4_)_2_ were dissolved into 175 mL distilled water and the solution was heated to *ca.* 80 °C. Then, 1.20 g of (NH_4_)_2_Fe(SO_4_)_2_ dissolved into 25 mL distilled water were added into FeNH_4_(SO_4_)_2_ solution, and the mixture was heated to ca. 80 °C with stirring. Next, 3.5 mL of NH_3_·H_2_O (25%) were poured into the solution with vigorous stirring. The black precipitate (Fe_3_O_4_) appeared immediately. The black precipitate was collected by magnet and washed with distilled water. Silica coating was done by using sodium metasilicate as follows. Sodium metasilicate (23.75 g) was dissolved into 200 mL distilled water, and ca. 20 mL hydrochloric acid solution (18.5 wt. %) were added to get the pH value of 12–13. The water dispersed Fe_3_O_4_ nanocores (in 400 mL) were dumped into sodium metasilicate solution with a mechanical stirrer, and the mixture was heated to 80 °C. The hydrochloric acid solution (18.5 wt. %) was then added dropwise to adjust the pH value to ca. 7. The Fe_3_O_4_@SiO_2_ was collected by magnet and washed several times with distilled water and centrifuged before dispersing into absolute ethanol (ca. 500 mL). Four milliliters of (3-Aminopropyl)triethoxysilane were added to the suspension, and the resulting mixture was mechanically stirred at room temperature for 85 h. The precipitate was collected by magnet and washed several times with ethanol, and then dispersed in ethanol (ca. 500 mL) as stock solution. Images of transmission electron microscopy (TEM) show particles with size of ~15–20 nm.

#### 2.3.2. Phthalocyanine-Functionalized Magnetic Silica Nanoparticles (MSNP-Pc1)

Previously prepared ethanol suspension of **MSNP** (11.5 mL, corresponding to 190 mg of MSNPs) was filtrated through a polyamide membrane, washed several times with DMSO, and resuspended in DMSO (1–2 mL). A solution of **Pc1** (15.0 mg, 12.9 µmol) in DMSO (4–5 mL) was added to the previous suspension, and the resulting mixture was stirred for 24 h at 150 °C. The immobilization of **Pc1** was monitored by thin-layer chromatography: the spot corresponding to **Pc1** decreases progressively while the spot of the hybrid **MSNP-Pc1** (at the application point) increased. The resulting hybrid material **MSNP-Pc1**, a green insoluble material, was washed several times with THF and then dichloromethane/methanol (90:10) until the Q band of the **Pc1** was no longer detected through UV–Vis in the rinse solvent. The amount of unreacted **Pc1** was calculated by UV–Vis spectrophotometry, based on the ε value at the Q band for **Pc1**, and it was determined that 2.9 mg of the 15.0 mg of **Pc1** starting material did not react. In the washing process, the hybrid material was firstly decanted on a magnetic field and then filtered under vacuum, using a polyamide membrane on the Büchner funnel. The hybrid material **MSNP-Pc1** was resuspended and kept in dry DMSO (25 mL). After catching the material with a magnet and decanting the supernatant, **MSNP-Pc1** was re-dispersed in 10 mL of pure ethanol; then, a stable suspension was formed. This suspension was used as stock solution of **MSNP-Pc1** (190 mg of MNSPs with 12.1 mg of **Pc1** grafted, re-suspended in 10 mL of ethanol) for the following studies. The particle size distribution in this suspension was investigated using dynamic light scattering (DLS). **UV–Vis** (DMSO) λ_max_: 705 nm. **UV–Vis** (solid-state) λ_max_: 410, 710 nm. **DLS:** Size between 90 and 250 nm with highest distribution at ca. 160 nm.

### 2.4. Anion Binding Studies

Anion binding studies were carried out by UV–Vis and fluorescence spectroscopic titrations in DMSO and water. To study the equilibrium between the Pc and the anions, titrations of **Pc1** (≈10^−6^ M) were performed by adding aliquots of an anion stock solution (≈10^−3^ M). After reaching the equilibrium, the absorbance (or the fluorescence) was measured. In the case of **MSNP-Pc1**, and to minimize the errors, a more concentrated stock solution (≈10^−3^ M) was used. In this case, aliquots of an anion solution 10 times more concentrated (≈10^−2^ M) were added. The corresponding binding isotherms, caused by the addition of the anions, were determined from the absorption and fluorescence variations at a selected wavelength. The binding isotherms were fitted assuming a non-linear regression analysis with 1:2 binding stoichiometry in agreement with Equation (1) [41]:

(1) $$\left(\Delta I/l)=([C]\times (K_{11.}\Delta \Phi_{11.}[A]+K_{11.}K_{12.}\Delta \Phi_{12.}{\left[A\right]}^{2})/(1+K_{11.}[A]+K_{11.}K_{12.}{\left[A\right]}^{2}\right)$$

where I is the fluorescence intensity, C is the chemosensor concentration, A is the anion concentration, and Φ_11_ and Φ_12_ are the constants associated with the emission quantum yields of the 1:1 and 1:2 complex formed, respectively. All measurements were done keeping the temperature at 22 °C. These measurements were made 2–3 times and reproducible within a 15–20% error range. The consistency between the calculated and experimentally observed binding profiles shows the evidence of the proposed 1:2 binding stoichiometry, following the same behavior as the previous published symmetric tetratosylamino Pc [40].

For the hybrid **MSNP-Pc1** a detail should be noted: the nanoparticles are dispersed in the solvent. However, the light scattering caused by this dispersion is linearly proportional in the range of the used concentration. To determine the affinity constants, it was assumed that this dispersion behaves as homogeneous solution.

## 3. Results and Discussion

### 3.1. Synthesis and Characterization

**Scheme 1:**
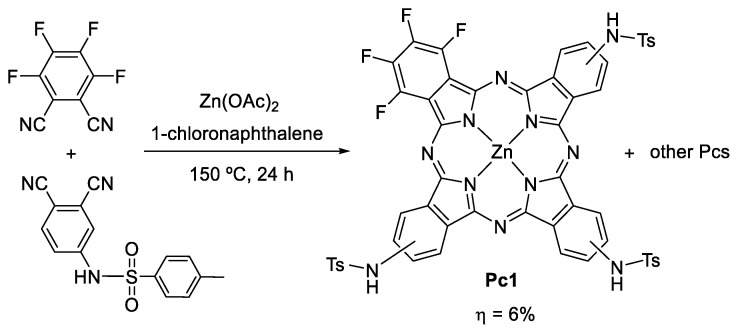
Synthesis of **Pc1**.

**Pc1** was prepared from the cross condensation of two phthalonitriles, 4-tosylaminophthalonitrile and tetrafluorophthalonitrile, in a 6% yield (Scheme 1). **Pc1** was characterized by UV–Vis (in solution and in solid-state); ^1^H, ^13^C, and ^19^F NMR spectroscopies; and HRMS (Figures S1–S4).

**Figure 1:**
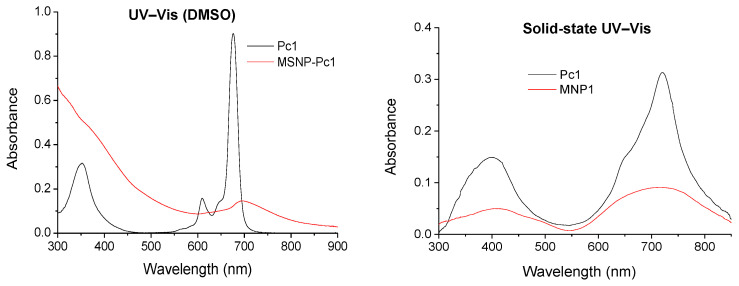
UV–Vis spectra in DMSO (left) and in solid-state (right) spectra of **Pc1** (black line) and **MSNP-Pc1** (red line).

The UV–Vis spectrum of **Pc1** shows a Soret band at 355 nm and a Q band at 680 nm, typical bands observed for metallated Pcs (Figure 1). The ^1^H NMR spectrum shows two multiplets at δ 7.91–8.04 and 8.91–9.08 ppm due to the resonances of the three *β*-H and the six *α*-H, respectively. The signals due to the resonance of the tosyl groups are present as a singlet (δ 2.26 ppm) and two multiplets (δ 7.36–7.46 and 7.91–8.04 ppm) due to the CH_3_ and aromatic (*ortho* and *meta*) protons, respectively. Finally, there is a singlet at δ 11.17 ppm due to the resonance of the NH protons. The ^13^C NMR spectrum confirms the presence of the tosyl groups due to the resonance of the CH_3_ carbons at δ 21.00 ppm. The ^19^F NMR shows two mutiplets at δ −177.84 to −174.50 and −165.98 to −163.06 ppm due to the *β*-F and *α*-F resonances, respectively. The HRMS showed a peak at *m*/*z* 1159.9999 corresponding to [M+4H]^+^. All these spectra support the proposed structure to **Pc1**.

**Scheme 2:**
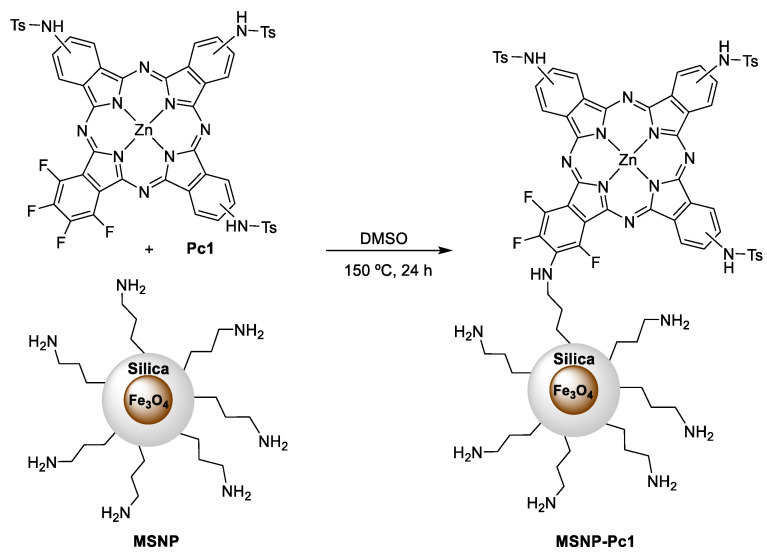
Synthesis of **MSNP-Pc1**.

The reaction of **Pc1** with the previously prepared ferromagnetic nanoparticles was performed following the methodology previously described by us [29], with minor modifications (Scheme 2). The covalent bonding was achieved by aromatic nucleophilic substitution of at least one of the fluorine atoms present in **Pc1** and the amino groups of the **MNSP**. The coupling reaction of **Pc1** on the magnetic nanomaterial **MNSP** was carried out in DMSO at 150 °C. After 24 h, the resulting solid material, **MSNP-Pc1**, was filtered and washed with the appropriate solvent in order to remove the residual unbound **Pc1**. This process was monitored by UV–Vis spectra and stopped when no Q band was detected in the rinse solvent. The amount of **Pc1** covalently bound to the nanomaterial was calculated by subtracting the amount of recovered Pc in the combined washing solvents (measured by UV–Vis) to the initial amount of **Pc1** used. The hybrid material **MSNP-Pc1** was then resuspended in ethanol and used in the following anion binding studies.

**Figure 2:**
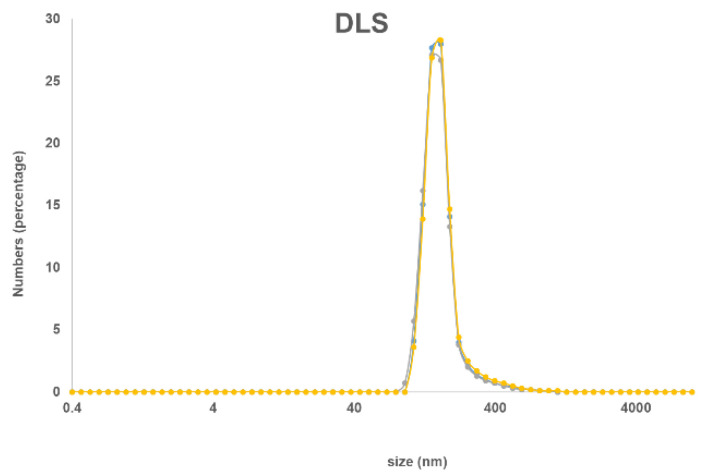
Dynamic light scattering (DLS) spectrum of **MSNP-Pc1** in ethanol.

The new nanohybrid material was characterized by solution and solid UV–Vis spectroscopy and by DLS. In the solid-state, the typical Soret and Q bands of the Pcs at 410 and 710 nm were observed (Figure 1), while in a DMSO suspension there is a small and very broad Q band around 705 nm. The size distribution of the nanohybrid material in suspension was measured by DLS with the highest distribution at 160 nm (Figure 2).

### 3.2. Anion Binding Studies

**Figure 3:**
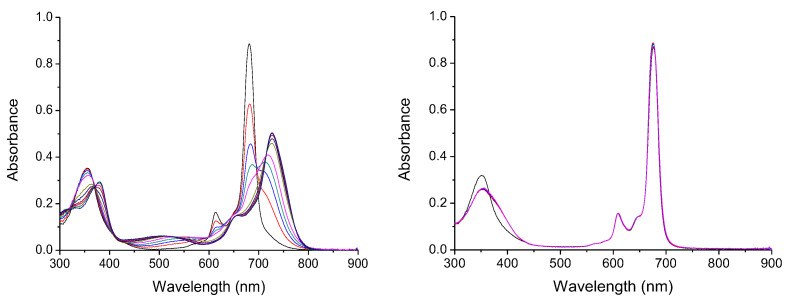
Absorption spectra of **Pc1** (5.4 × 10^−6^ mol.dm^−3^) in DMSO upon addition of increasing concentration of CN− (5.4 × 10^–6^ mol.dm^–3^) (**left**) and Cl− (4.3 × 10^–3^ mol.dm^–3^) (**right**).

To develop a new, effective, and applicable sensor for anions, it is mandatory that they have host–guest interactions in aqueous media. Since **Pc1** is not water soluble, the anion biding studies were performed in DMSO. DMSO is a highly competitive solvent that interacts through hydrogen bonds in the available binding sites, behaving similarly to water. Significant changes in the UV–Vis spectra of **Pc1** were observed upon addition of the anions AcO−, CN−, F−, H_2_PO_4_−, NO_2_−, and OH− (as TBA salts solutions). In these conditions, it is evident that **Pc1** decreases its absorption intensity, and there is a bathochromic shift of the Soret and Q bands, being more relevant for the last ones (Figure 3 and Section S2.1). However, with the addition of Br−, Cl−, HSO_4_−, and NO_3_− (as TBA salts), no significant changes were observed in the UV–Vis spectra (Figure 3 and Section S2.1). This observed bathochromic shift occurs due to the formation of a new conformation of the Pcs in solution. The new conformation occurs due to the interaction of anions with tosylamino groups and the zinc ion in the Pc core, through the formation of hydrogen bonds and co-axial coordination [40]. These results are in line with our previous work, where symmetrical phthalocyanines containing four tosylamino groups demonstrated a high sensitivity for several anions, in special to CN− [40].

**Figure 4:**
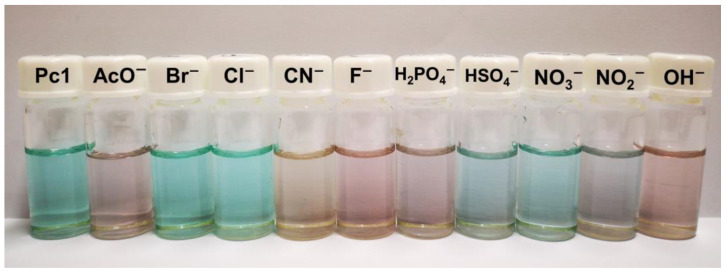
Changes in the color solutions of **Pc1** in DMSO upon the addition of different anions (as TBA salts) in DMSO.

During the titrations of the chemosensor **Pc1**, a drastic change was noticed in the solution color, from green to violet in the presence of AcO−, F−, H_2_PO_4_−, and OH−, to blue when in the presence of NO_2_− and to the most distinct and notorious a pale-yellow, when in the presence of CN− (Figure 4). These color changes confirm the ability of **Pc1** to act as optical chemosensor.

A similar anion binding study was performed with the new hybrid material **MSNP-Pc1** and all the previous anion solutions, both in DMSO suspensions (Section S2.2) and in water (Section S2.3). However, a minimal bathochromic shift was observed for most of the studied anions. To prevent possible miscalculations in the determination of the anion binding constants, fluorescence spectroscopy was used to infer the interaction ability of the new material with anions.

**Figure 5:**
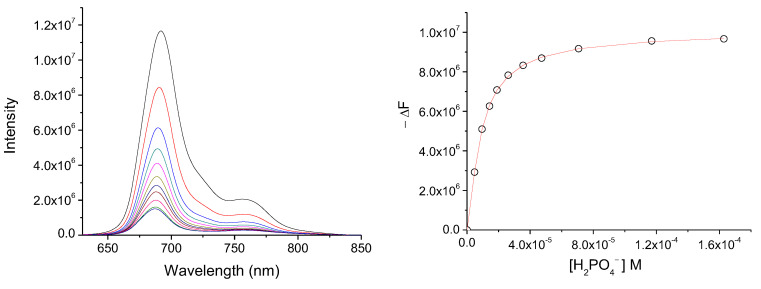
Fluorescence spectra of **Pc1** (5.4 × 10^−6^ mol.dm^−3^) in DMSO upon addition of increasing concentration of H_2_PO_4_− (7.1 × 10^−3^ mol.dm^−3^) when excited at 600 nm (**left**); and the binding profiles and the generated fit at 688 nm (**right**).

To compare the experimental results, both **Pc1** and **MSNP-Pc1** were studied through fluorescence using DMSO (for **Pc1** and **MSNP-Pc1**) and H_2_O (for **MSNP-Pc1**) as solvents. The addition of anions to **Pc1** solution induced a decrease in the fluorescence intensity (Figure 5 and Section S3.1).

As expected, with the addition of the anions to the **MSNP-Pc1** suspension provoked similar alterations in the **MSNP-Pc1** spectra (Sections S3.2 and S3.3). The anion binding constants were determined by fitting Equation (1) to the plot of the fluorescence intensity variation against the concentration of the anions (Table 1).

The values determined for the anion binding constants indicate that **Pc1** is able to interact strongly with the several anions under study. These binding constants are actually much higher than those found for the related tetra-tosylamino and octa-tosylamino Pcs, reported by us in previous works [39,40]. Standard methods for anions detection such as flow injection, potentiometry, electrochemical, and polarography are time-consuming. Consequently, an increasing demand for the development of more efficient, selective, and sensitive methods to measure harmful anions, at the scale of microgram/liter level, is required. Thus, fluorescent chemosensors for harmful anions are significantly attractive due to their low cost and numerous advantages, including high sensitivity, low limits of detection (LoD), and easy operation. The LoD is commonly used as evidence of the quality of a chemosensor. The LoD for **Pc1** and **MNSP-Pc1**, in DMSO and water, were calculated using Equation (2) [42]:

LoD = 3**S**/**ρ** (2)

where **S** is the standard deviation of blank measurements (10 runs) and **ρ** is the slope between intensity versus sample concentration. Table 2 presents the LoD of each anion for **Pc1** and **MNSP-Pc1**.

Comparing the obtained results for **Pc1** with those previously reported [39,40], it is possible to conclude that the removal of one tosylamino group in the macrocycle induces an increased affinity for anions, which is explained by the decrease of steric hindrance in the network complex formation. Nevertheless, it is also clear that, decreasing the number of tosylamino moieties, the binding sites, in the Pcs core, leads to a decrease on the specificity of the chemosensor: the affinity constants found for **Pc1** are at the same order for all anions studied.

**Figure 6:**
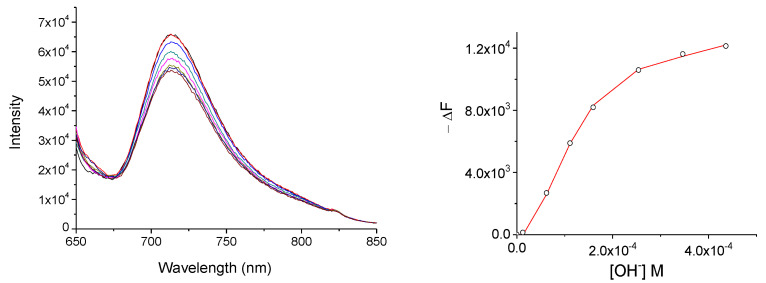
Fluorescence spectra of **MSNP-Pc1** (2.4 × 10^−4^ mol.dm^−3^) in H_2_O upon increasing concentration of OH− (7.5 × 10^−3^ mol.dm^−3^) when excited at 630 nm (**left**); and binding profiles and the generated fit at 725 nm (**right**).

In the case of the **MSNP-Pc1**, as previously stated, the hybrid is in suspension and that may hinder the chemosensor:anion equilibrium, decreasing the affinity in both DMSO and water. The results show that **MSNP-Pc1** interacts with most of the anions studied in water (Figure 6 and Section S3.3), enabling the quantification for cyanide and hydroxide anions. As referred, a chemosensor for anions should be effective in water.

### 3.3. Reversibility and Reusability Studies

**Figure 7:**
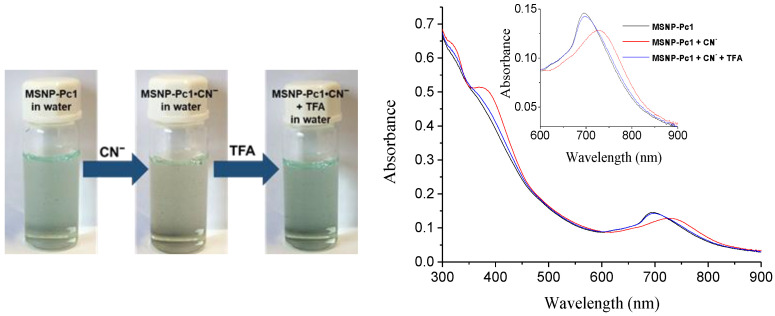
Colorimetric response of **MSNP-Pc1** for reversibility and reusability tests with CN− ion (**left**). UV–Vis spectral changes of the complex **MSNP-Pc1**•CN− treated with a TFA water solution (5.0 × 10^−3^ mol.dm^−3^) (**right**).

The reversibility and reusability of the new material **MSNP-Pc1**, without losing its efficiency, are very important properties for chemosensing or cleaning applications. To evaluate those properties, the complex **MSNP-Pc1•CN−** was prepared by adding cyanide anion (5.0 × 10^−3^ mol.dm^−3^) in water, and the process was monitored by UV–Vis (Figure 7). Then, successive additions of 10 μL aliquots of an aqueous TFA solution (5.0 × 10^−3^ mol.dm^−3^) were done to the complex **MSNP-Pc1•CN−** until complete regeneration of the **MSNP-Pc1** chemosensor. The visual inspection also allowed following the chemosensor regeneration; the color of the suspension returned to the initial point after the addition of TFA (Figure 7). This study confirms that acid treatment of the **MSNP-Pc1•CN−** adduct immediately restores/regenerates the chemosensor **MSNP-Pc1**, with the consequent colorimetric change to the initial color.

**Figure 8:**
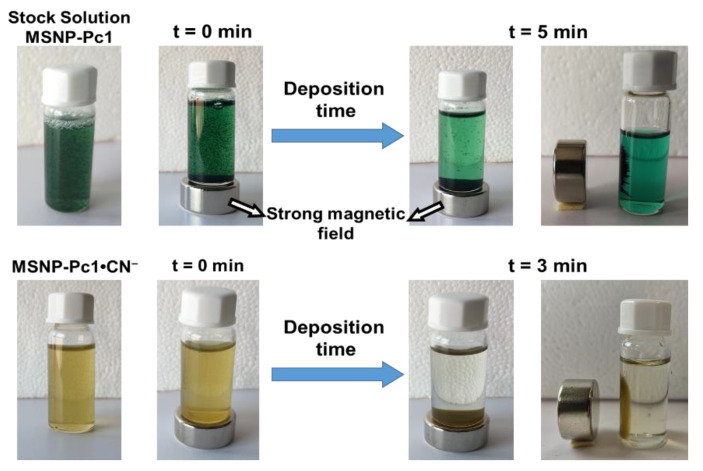
Water suspensions of **MSNP-Pc1** and **MSNP-Pc1**•CN− in the absence and presence of a strong magnetic field, with their corresponding deposition times.

To confirm the reusability of **MSNP-Pc1**, cyanide (5.0 × 10^−3^ M) was added to water suspension of **MSNP-Pc1**. This addition induced a color change. Then, TFA solution (5.0 × 10^−3^ M in water) was added. After 5 min stirring, a magnetic field was applied to the suspension (Figure 8), the solution was decantated, and the deposited material **MSNP-Pc1** was recovered. The material was re-suspended and CN− or OH− solutions were added. The new affinity constants (≈1.5 × 10^8^ and ≈7 × 10^7^, for CN− and OH−, respectively) were similar to those obtained with the “original” **MSNP-Pc1** (Table 1). This process was repeated five times without significant loss of anion sensibility.

## 4. Conclusions

A molecular optical chemosensor based on a tri(tosylamino)phthalocyanine (**Pc1**) was shown to be very effective to detect anions in DMSO and water. Remarkably, the hybrid material resulting from the immobilization of **Pc1** onto MSNPs (**MSNP-Pc1**) revealed an increased sensitivity and specificity for F−, CN−, and OH− in DMSO and for CN− and OH− in water. This hybrid material fulfills important requirements in the development of new chemosensors: (i) it has high affinity to some harmful anions; (ii) it can be easily recovered by applying a magnetic field; and (iii) it is reusable without loss of the sensing ability. In conclusion, our results show that MSNPs decorated with adequately functionalized Pcs act as chemosensors in both organic and aqueous media. This novel hybrid can also be explored as a removal agent for pollutant anions, such as CN−, from contaminated waters.

## Figures and Tables

**Table 1:** Anions binding constants *K* (M^−1^) found for **Pc1** and **MSNP-Pc1** in DMSO and water.

	Pc1	MSNP-Pc1	
Anion	*K*_DMSO_	*K*_DMSO_	*K*_Water_
AcO−	3.65 × 10^10^	5.50 × 10^7^	*n.d*
CN−	3.41 × 10^10^	8.75 × 10^8^	1.47 × 10^8^
F−	6.87 × 10^10^	2.42 × 10^9^	*n.d*
H_2_PO_4_−	3.32 × 10^10^	5.72 × 10^7^	*n.d*
NO_2_−	4.18 × 10^10^	*n.d*	*n.d*
OH−	6.35 × 10^10^	1.13 × 10^9^	6.73 × 10^7^

*n.d.*, non-determined, the variation in the fluorescence spectrum induced by the addition of the anion was not significant.

**Table 2:** Limit of detection (µM) for **Pc1** and **MSNP-Pc1** in DMSO and water.

	Pc1	MSNP-Pc1	
Anion	LoD _DMSO_	LoD _DMSO_	LoD _Water_
AcO−	5.00	4.59	–
CN−	5.55	5.04	0.278
F−	3.33	3.12	–
H_2_PO_4_−	4.76	4.22	–
NO_2_−	7.17	–	–
OH−	3.69	3.31	0.125

## Data Availability

Not applicable.

## Supplementary Materials

The following are available online at https://www.mdpi.com/1424-8220/21/5/1632/s1.
