# Supplementary material for: Phthalocyanine-Functionalized Magnetic Silica Nanoparticles as Anion Chemosensors
Source: Sensors (Basel). 2021 Feb 26;21(5):1632. doi: 10.3390/s21051632 (PMC7956555; doi:10.3390/s21051632)
Supplement: Supplementary file 1 [file sensors-21-01632-s001.pdf]

Supporting Information for

Phthalocyanine-functionalized magnetic silica nanoparticles as anion chemosensors

João M. M. Rodrigues,[1,*] Andreia S. F. Farinha,[2,*] Zhi Lin,[3] José A. S. Cavaleiro,[1] Augusto C. Tomé [1] and João P. C. Tomé [4,*]

[1]LAQV-REQUIMTE, Department of Chemistry, University of Aveiro, 3810-193 Aveiro, Portugal
[2]Water Desalination and Reuse Center (WDRC), Division of Biological and Environmental Sciences (BESE), King Abdullah University of Science and Technology (KAUST) Thuwal, 23955-6900, Saudi Arabia
[3]CICECO and Department of Chemistry, University of Aveiro, 3810-193 Aveiro, Portugal
[4]CQE, Departamento de Engenharia Química, Instituto Superior Técnico, Universidade de Lisboa, 1049-001 Lisboa, Portugal
* Correspondence: jrodrigues@ua.pt; andreia.farinha@kaust.edu.sa; jtome@tecnico.ulisboa.pt

Index

1 NMR, MS and TEM image of the new compounds 2

2 UV-Vis titration of **Pc1** and **MSNP-Pc1** with different anions 5

2.1 Titration of **Pc1** with anions (tetrabutylammonium salts) in DMSO 5

2.2 Titration of **MSNP-Pc1** with anions (tetrabutylammonium salts) in DMSO 8

2.3 Titration of **MSNP-Pc1** with anions (tetrabutylammonium salts) in water 12

3 Fluorescence titrations of **Pc1** and **MSNP-Pc1** with different anions 16

3.1 Titration of **Pc1** with anions (tetrabutylammonium salts) in DMSO 16

3.2 Titration of **MSNP-Pc1** with anions (tetrabutylammonium salts) in DMSO 19

3.3 Titration of **MSNP-Pc1** with anions (tetrabutylammonium salts) in water 23

1. NMR, MS and TEM image of the new compounds

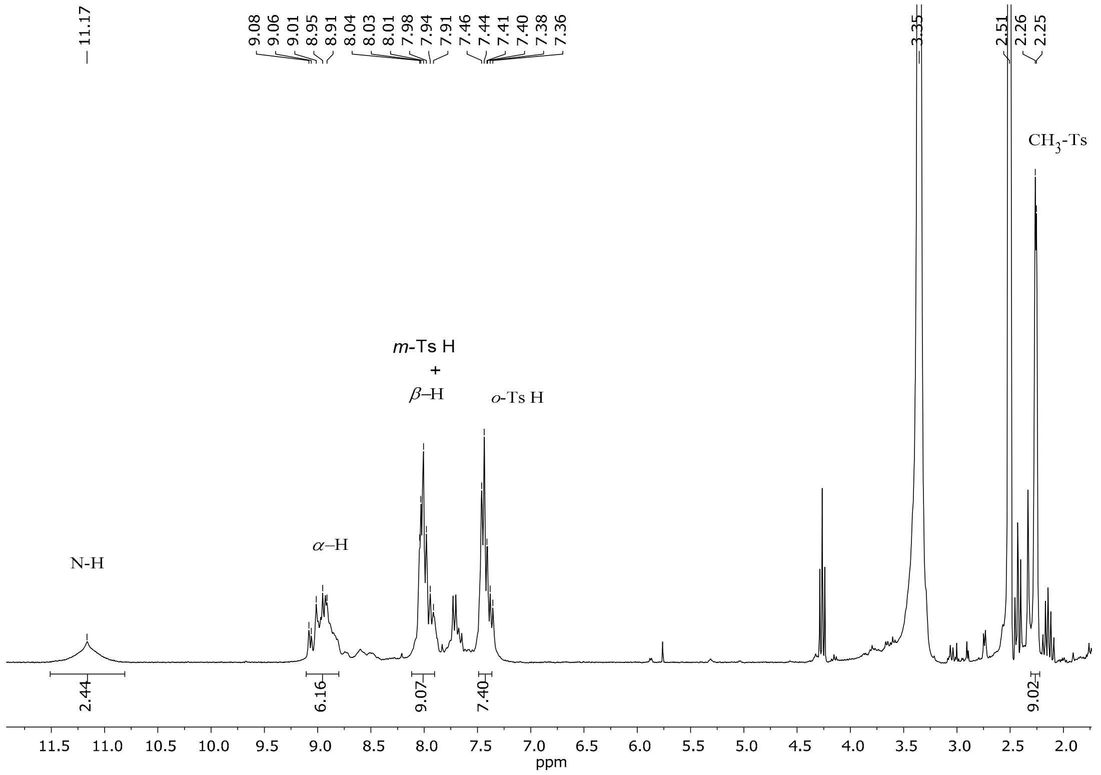

Figure S1. ^{1}H NMR spectrum of **Pc1** in DMSO-*d6.*

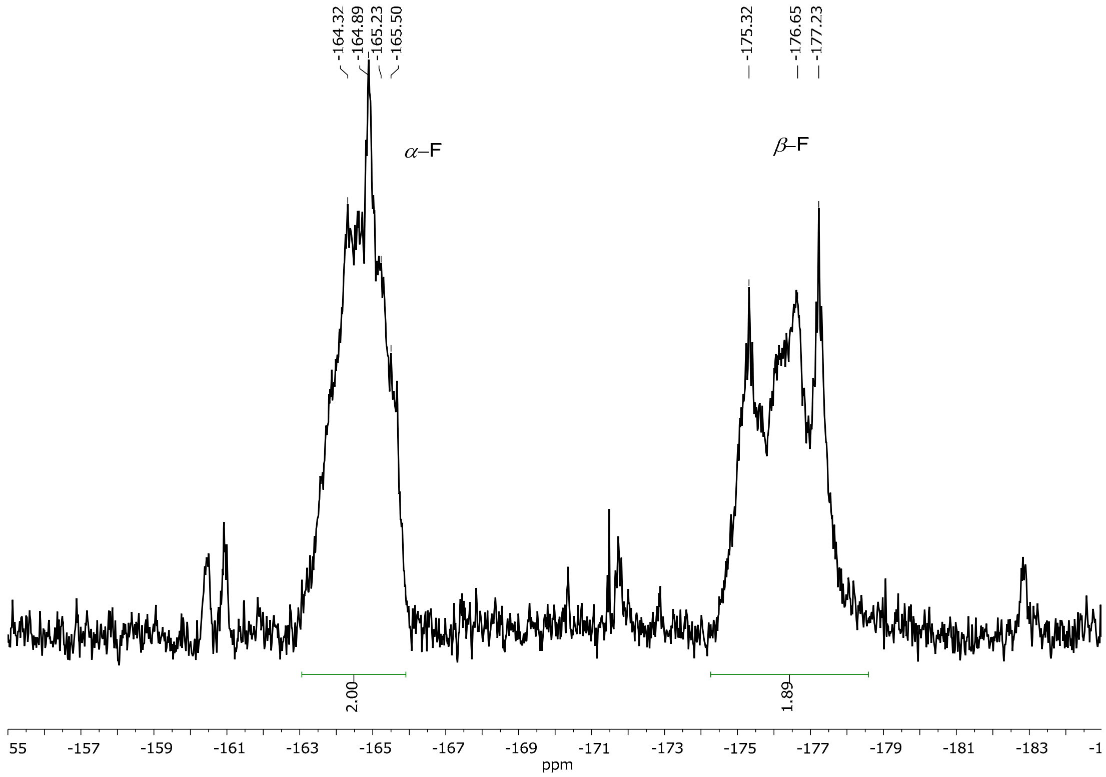

Figure S2. ^{19}F NMR spectrum of **Pc1** in DMSO-*d6.*

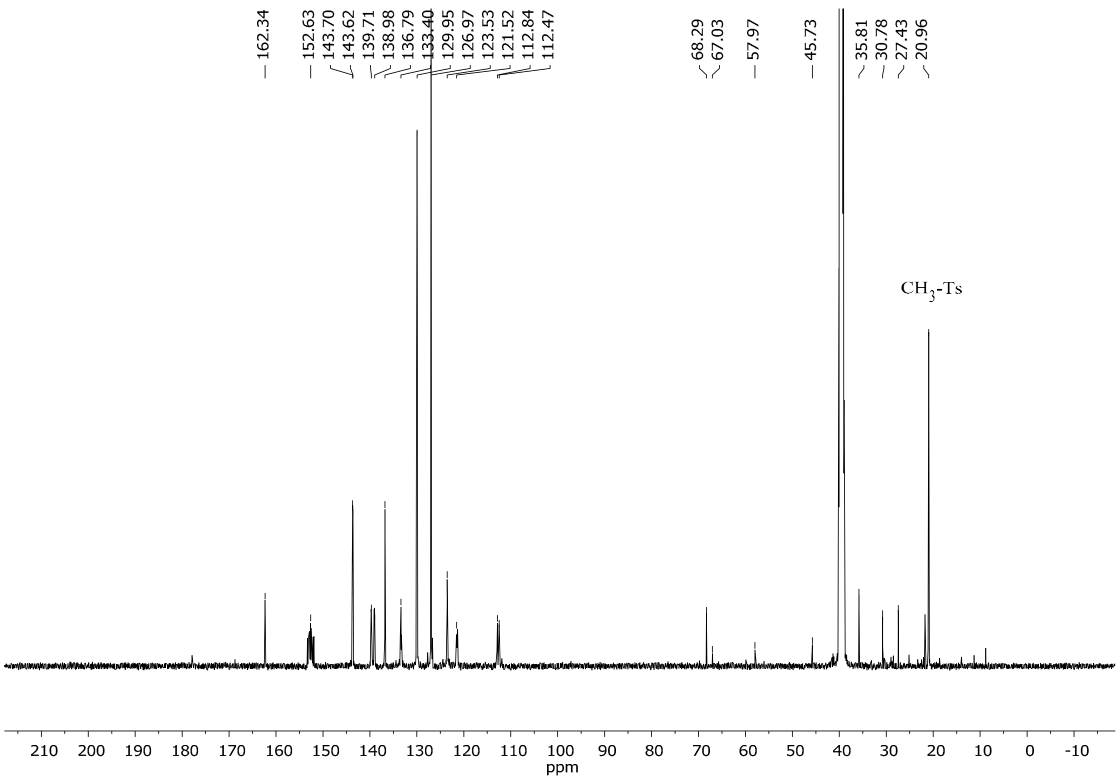

Figure S3. ^{13}C NMR spectrum of **Pc1** in DMSO-*d6.*

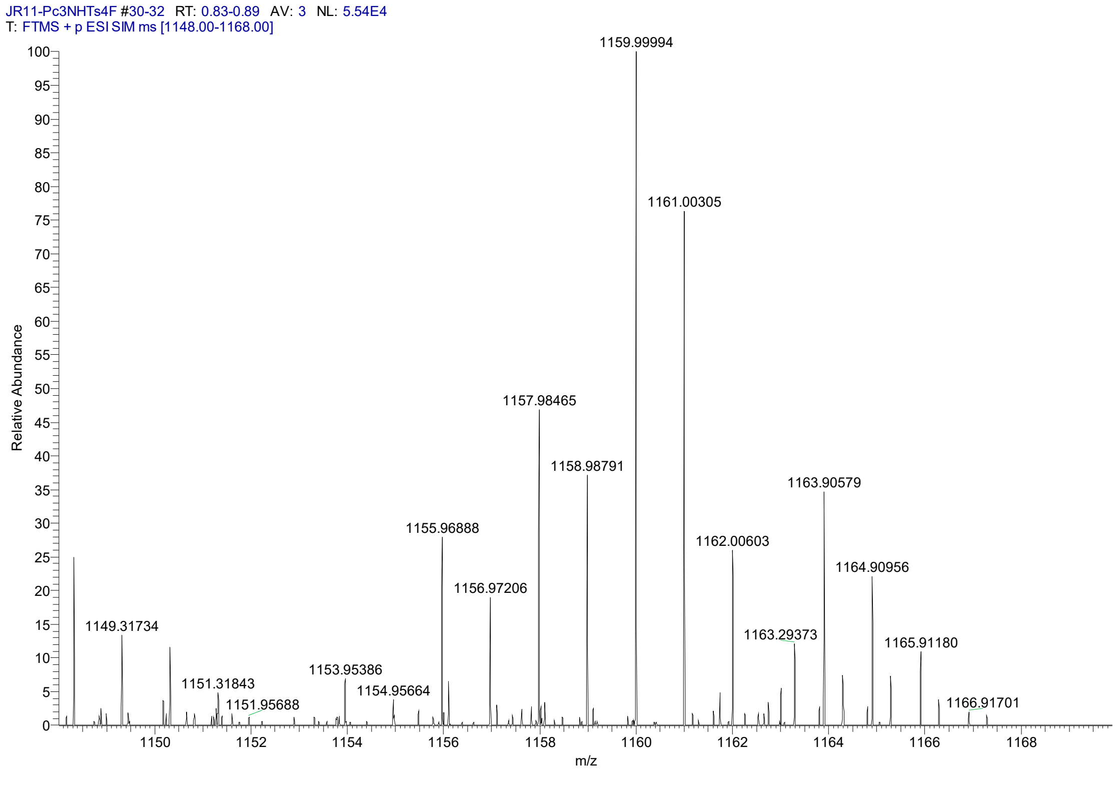

Figure S4. High resolution electrospray ionization mass spectrum (ESI MS) of **Pc1**.

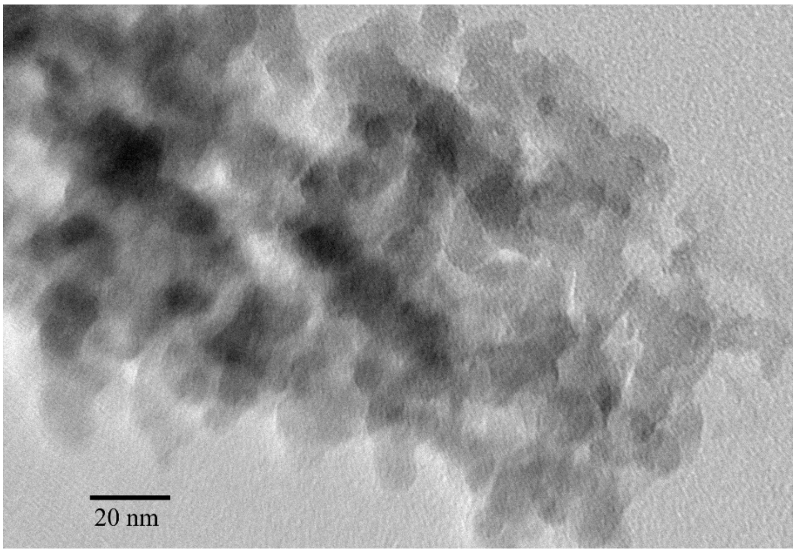

Figure S5. Transmission electron microscopy (TEM) of **MSNP**.

2. UV-Vis titration of Pc1 and MSNP-Pc1 with different anions

*2.1. Titration of **Pc1** with anions (tetrabutylammonium salts) in DMSO*

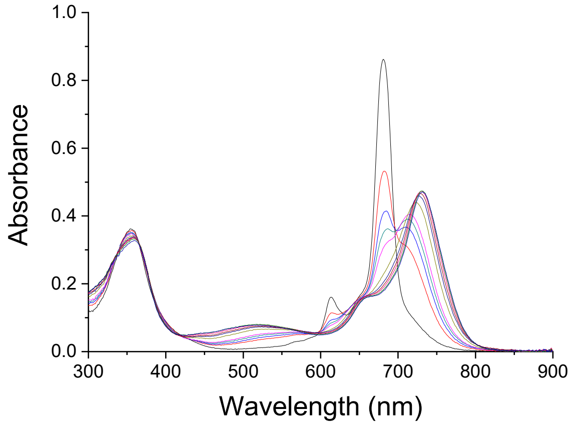

Figure S6. Titration of **Pc1** with acetate anion.

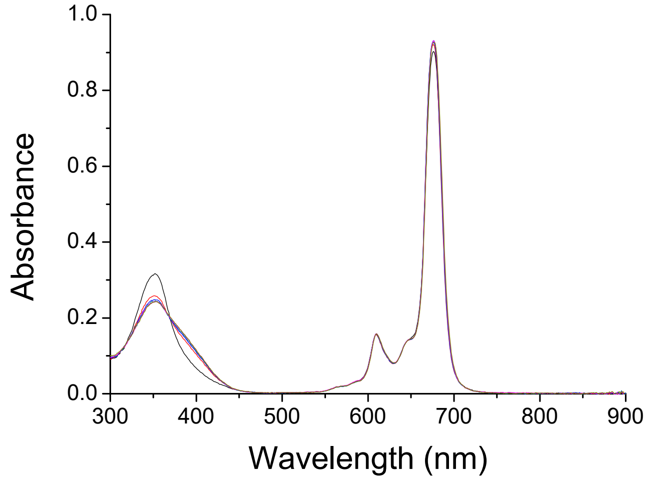

Figure S7. Titration of **Pc1** with bromide anion.

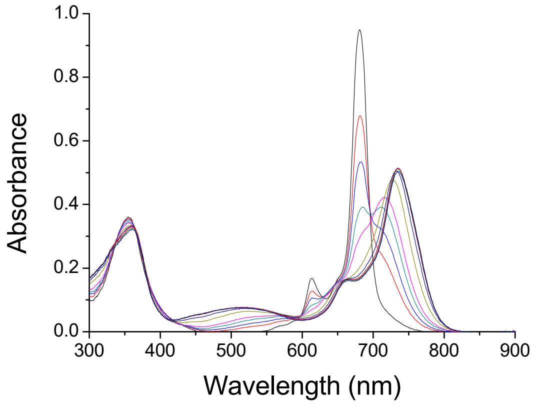

Figure S8. Titration of **Pc1** with fluoride anion.

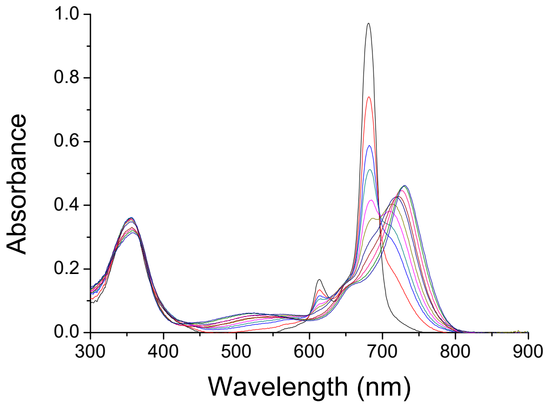

Figure S9. Titration of **Pc1** with dihydrogen phosphate anion.

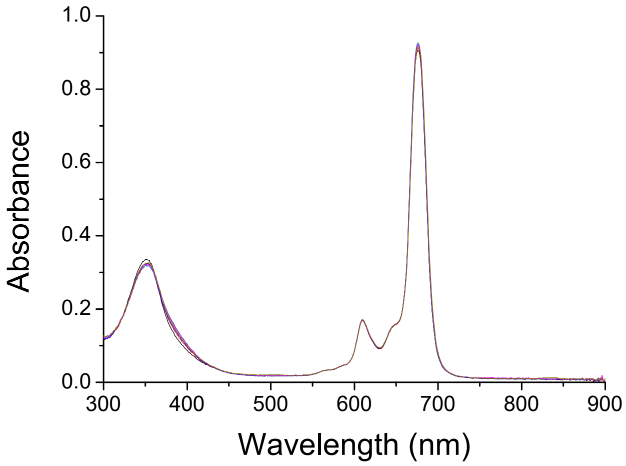

Figure S10. Titration of **Pc1** with hydrogen sulfate anion.

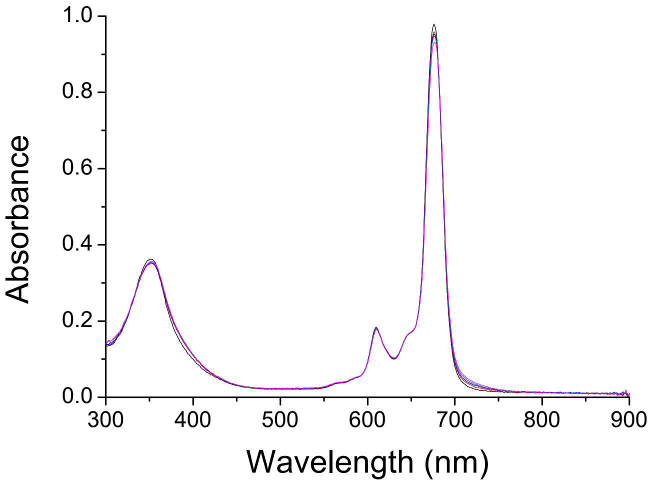

Figure S11. Titration of **Pc1** with nitrate anion.

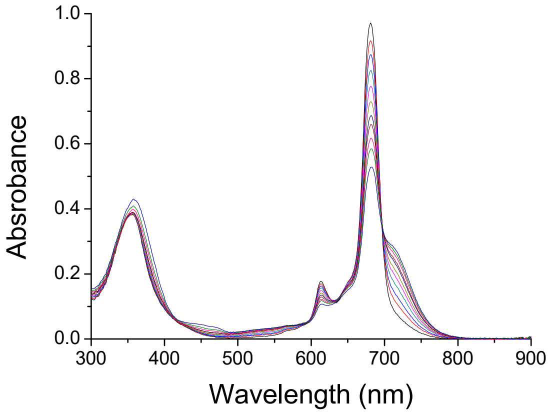

Figure S12. Titration of **Pc1** with nitrite anion.

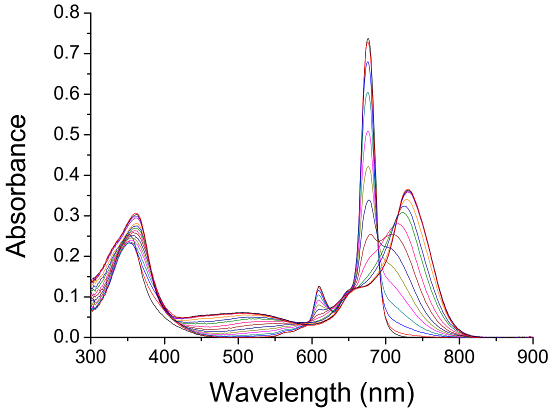

Figure S13. Titration of **Pc1** with hydroxide anion.

*2.2. Titration of **MSNP-Pc1** with anions (tetrabutylammonium salts) in DMSO*

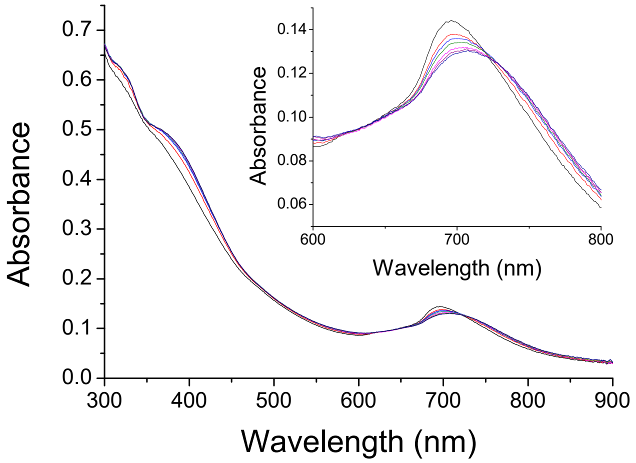

Figure S14. Titration of **MSNP-Pc1** with acetate anion.

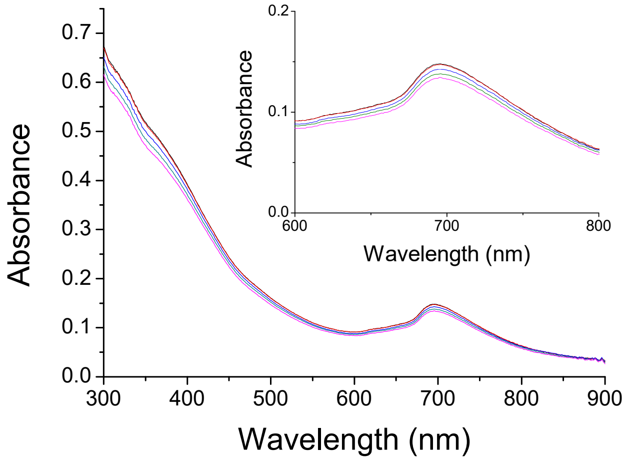

Figure S15. Titration of **MSNP-Pc1** with bromide anion.

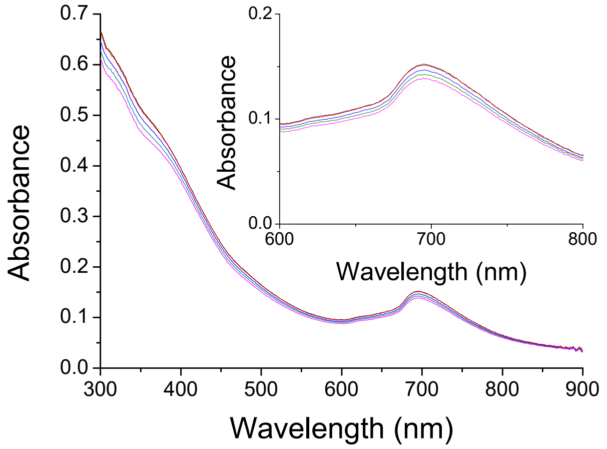

Figure S16. Titration of **MSNP-Pc1** with chloride anion.

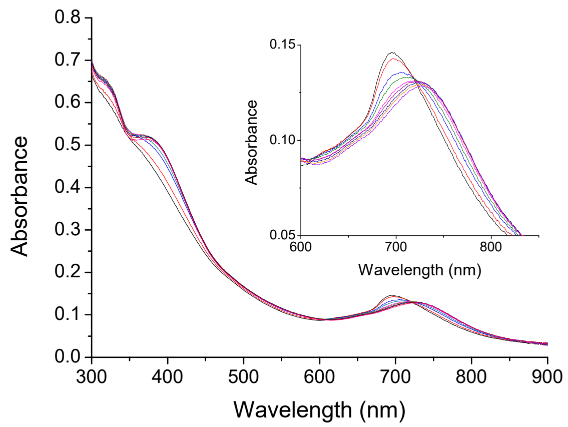

Figure S17. Titration of **MSNP-Pc1** with cyanide anion.

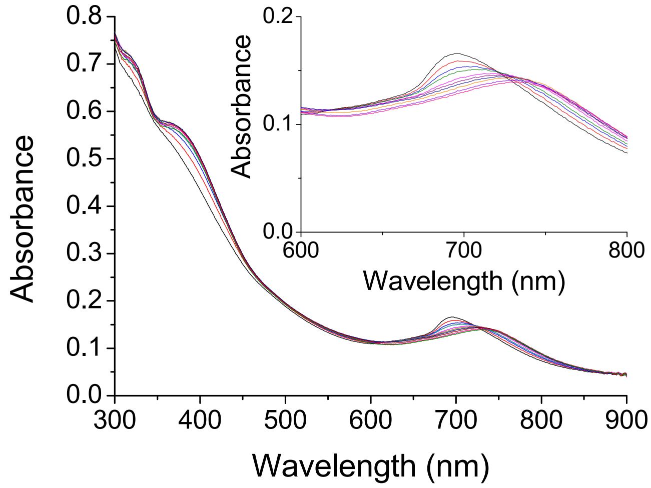

Figure S18. Titration of **MSNP-Pc1** with fluoride anion.

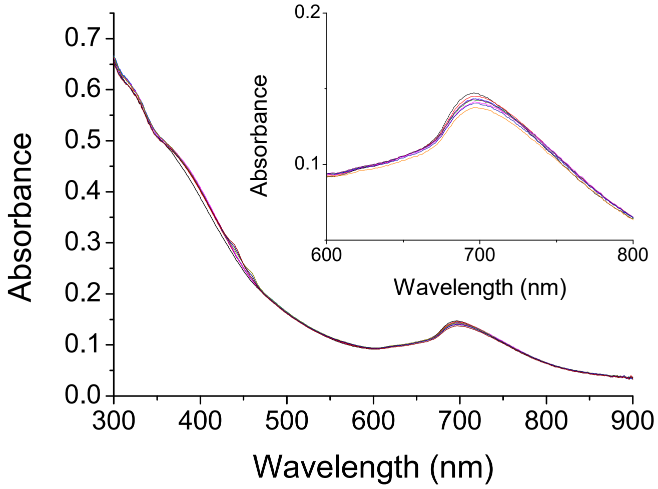

Figure S19. Titration of **MSNP-Pc1** with dihydrogen phosphate anion.

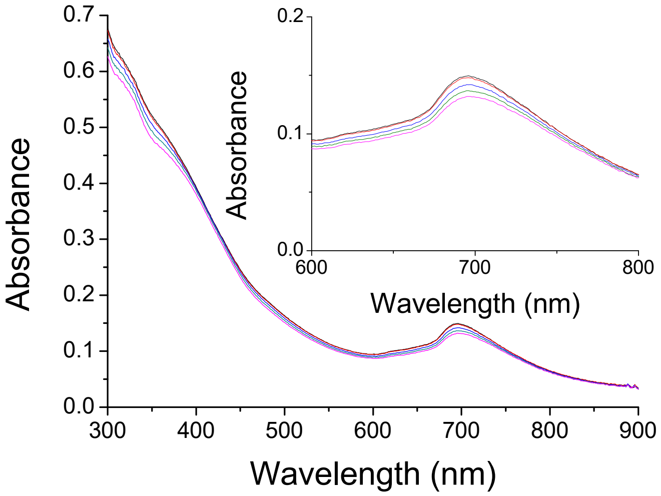

Figure S20. Titration of **MSNP-Pc1** with hydrogen sulfate anion.

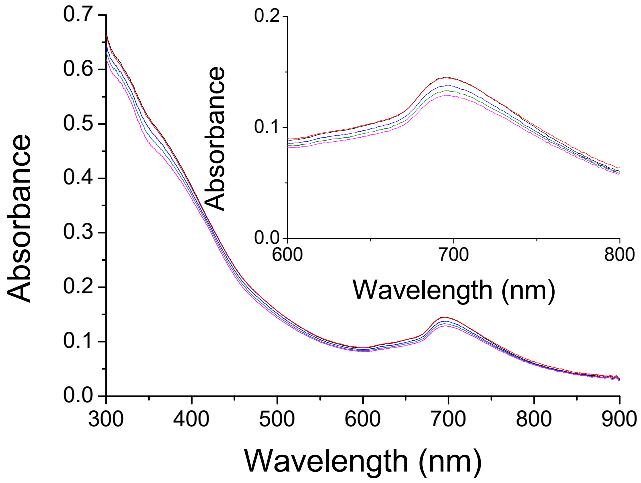

Figure S21. Titration of **MSNP-Pc1** with nitrate anion.

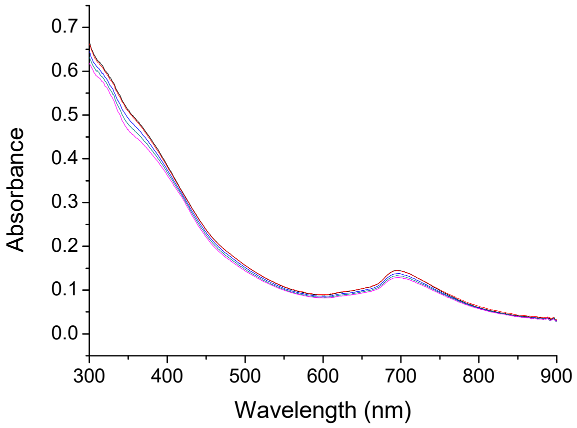

Figure S22. Titration of **MSNP-Pc1** with nitrite anion.

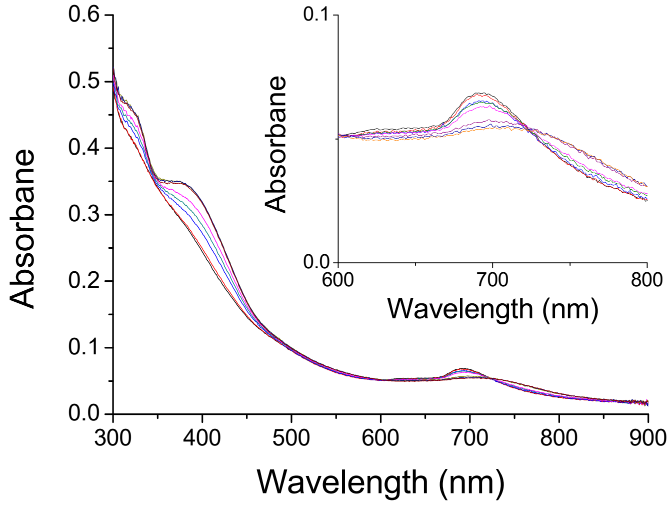

Figure S23. Titration of **MSNP-Pc1** with hydroxide anion.

*2.3. Titration of **MSNP-Pc1** with anions (tetrabutylammonium salts) in water*

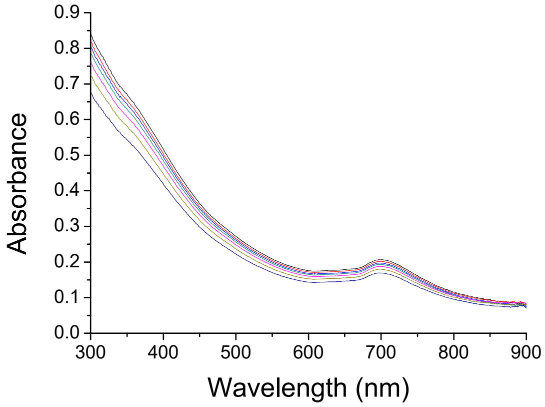

Figure S24. Titration of **MSNP-Pc1** with acetate anion.

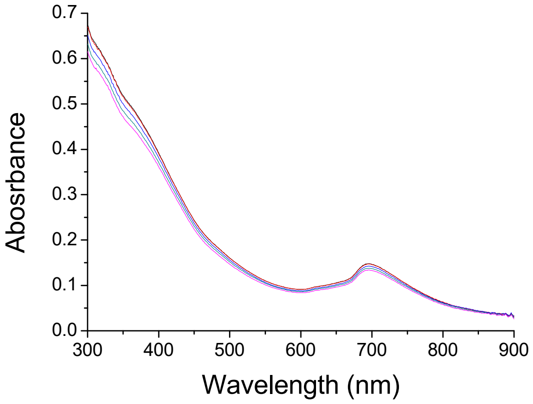

Figure S25. Titration of **MSNP-Pc1** with bromide anion.

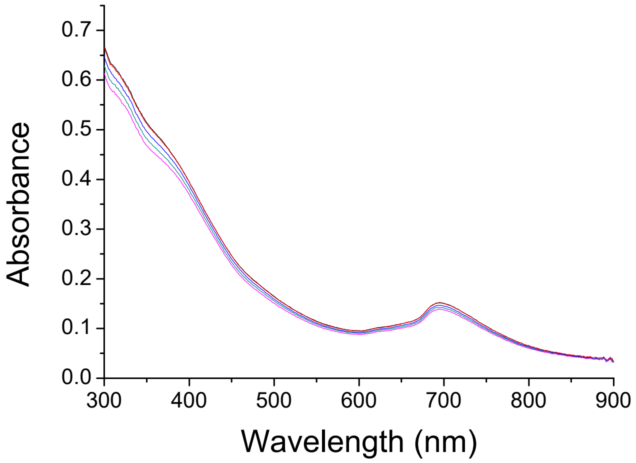

Figure S26. Titration of **MSNP-Pc1** with chloride anion.

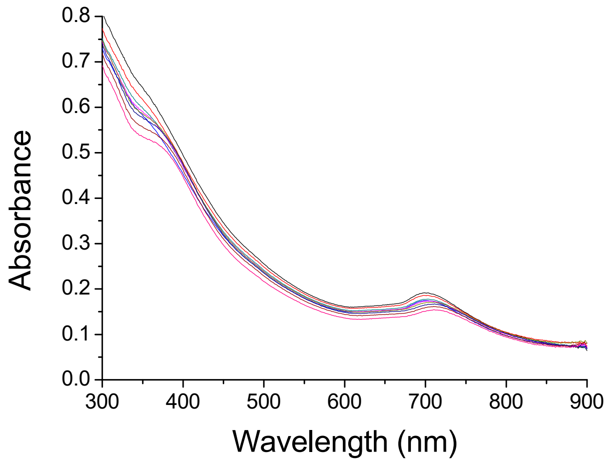

Figure S27. Titration of **MSNP-Pc1** with cyanide anion.

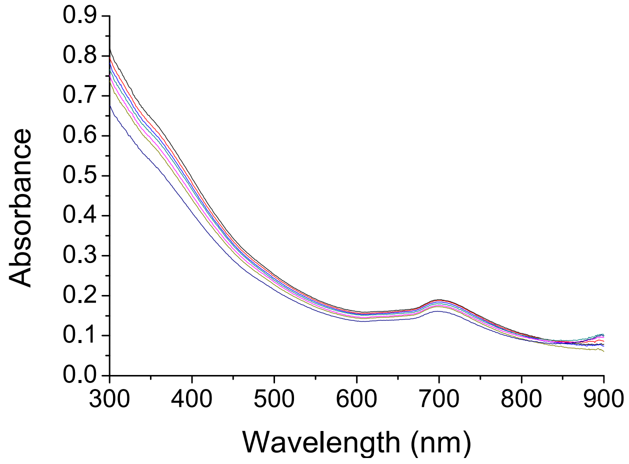

Figure S28. Titration of **MSNP-Pc1** with fluoride anion.

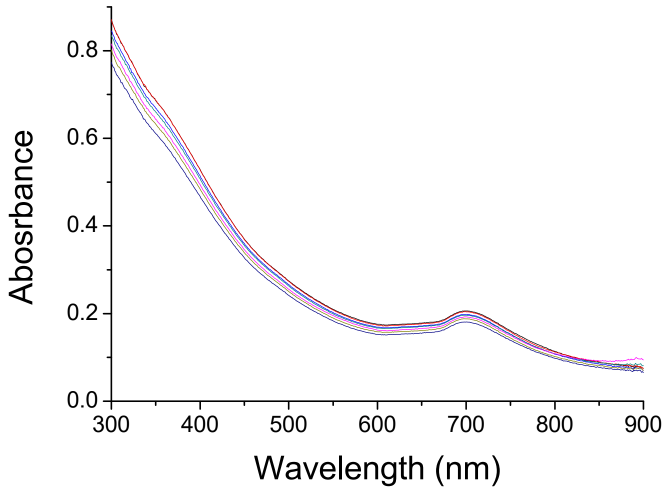

Figure S29. – Titration of **MSNP-Pc1** with dihydrogen phosphate anion.

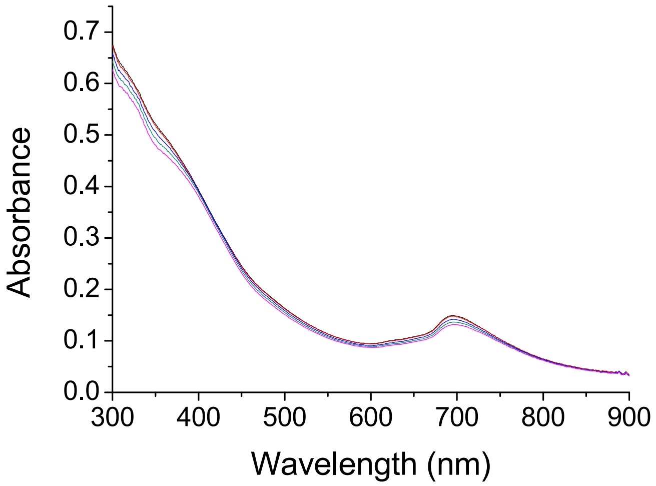

Figure S30. Titration of **MSNP-Pc1** with hydrogen sulfate anion.

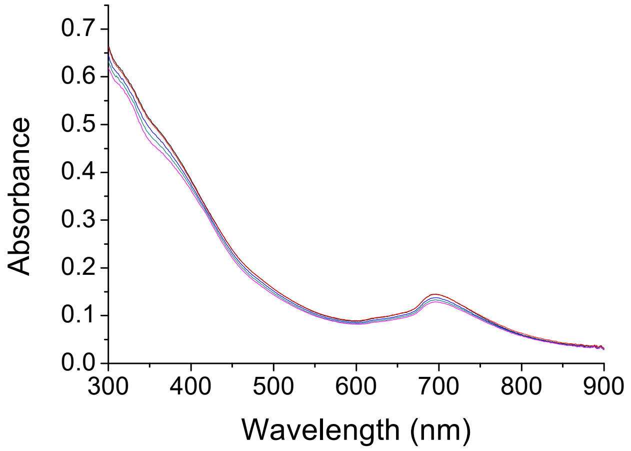

Figure S31. Titration of **MSNP-Pc1** with nitrate anion.

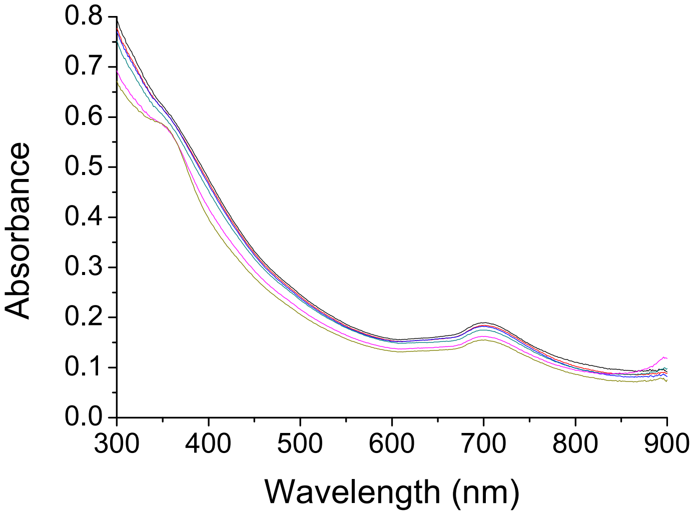

Figure S32. Titration of **MSNP-Pc1** with nitrite anion.

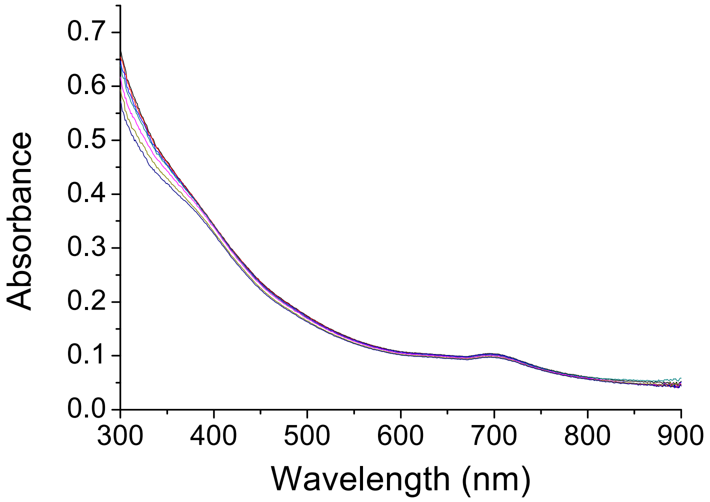

Figure S33. Titration of **MSNP-Pc1** with hydroxide anion.

3. Fluorescence titrations of Pc1 and MSNP-Pc1 with different anions

*3.1. Titration of **Pc1** with anions (tetrabutylammonium salts) in DMSO*

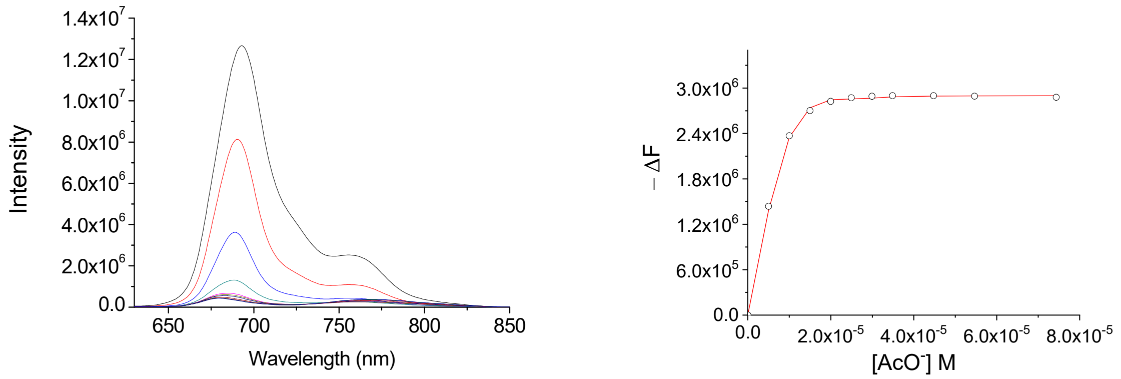

Figure S34. Titration of **Pc1** with acetate anion (left), and the plot of the experimental data and corresponding fit to a 1:2 binding model (right).

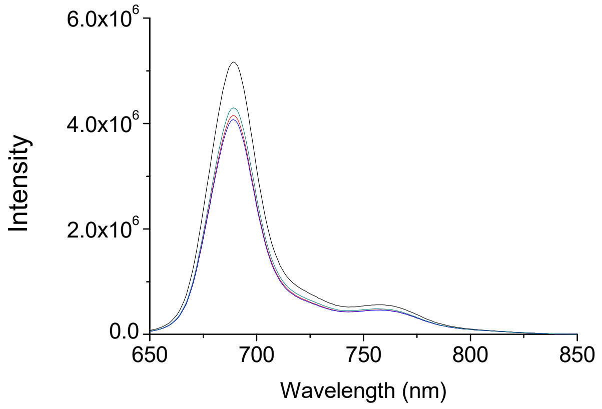

Figure S35. Titration of **Pc1** with bromide anion.

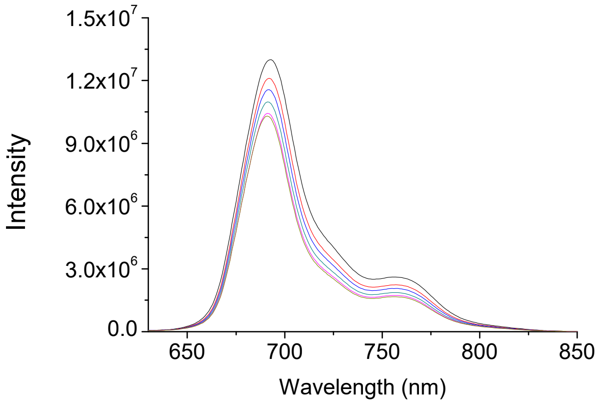

Figure S36. Titration of **Pc1** with chloride anion.

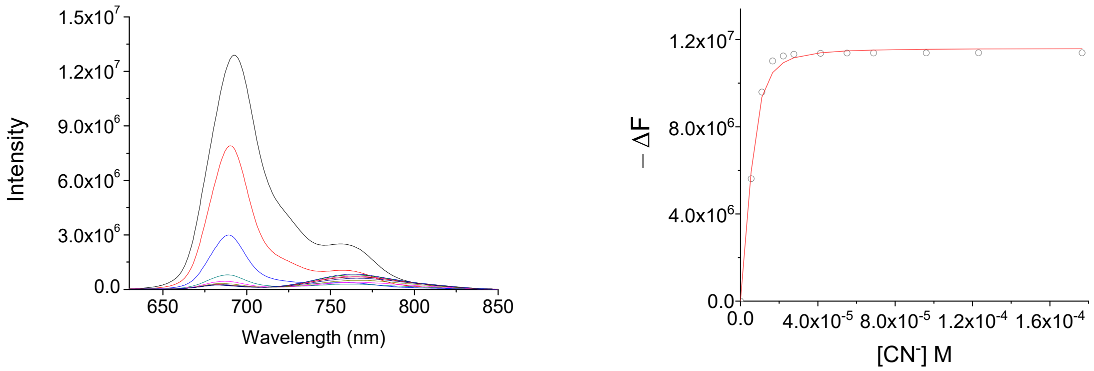

Figure S37. Titration of **Pc1** with cyanide anion (left), and the plot of the experimental data and corresponding fit to a 1:2 binding model (right).

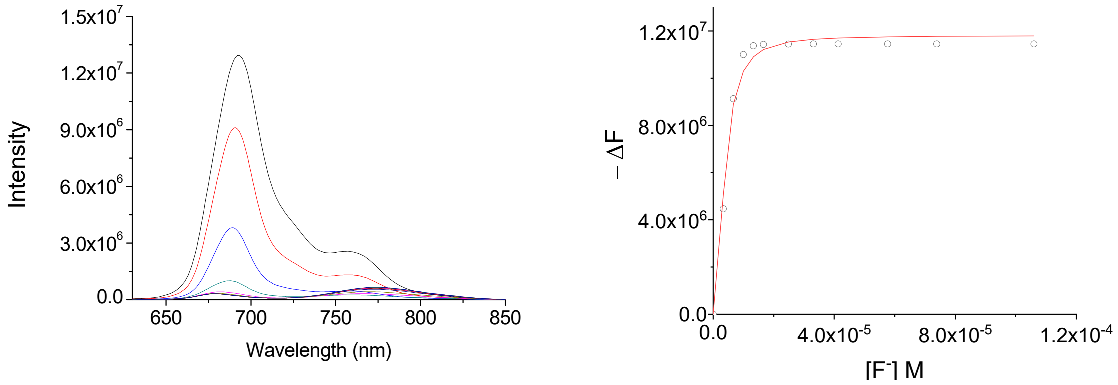

Figure S38. Titration of **Pc1** with fluoride anion (left), and the plot of the experimental data and corresponding fit to a 1:2 binding model (right).

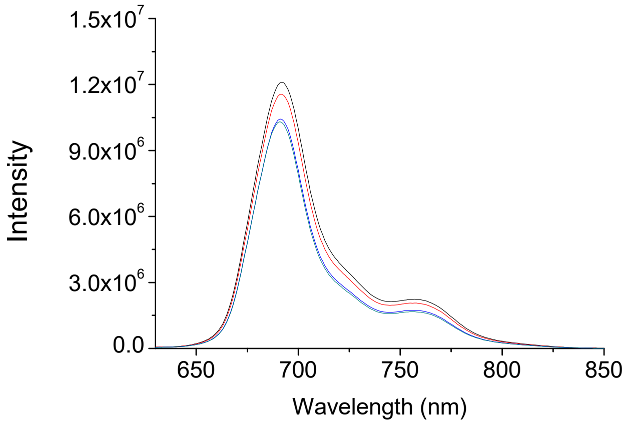

Figure S39. Titration of **Pc1** with hydrogen sulfate anion.

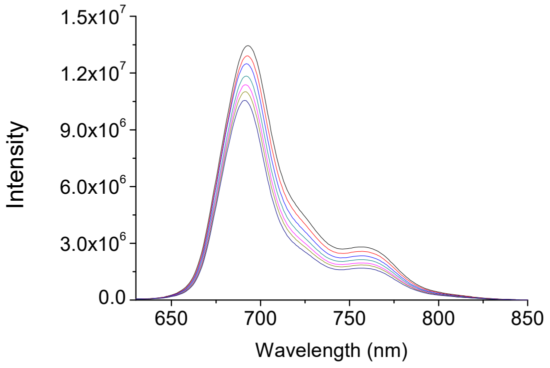

Figure S40. Titration of **Pc1** with nitrate anion.

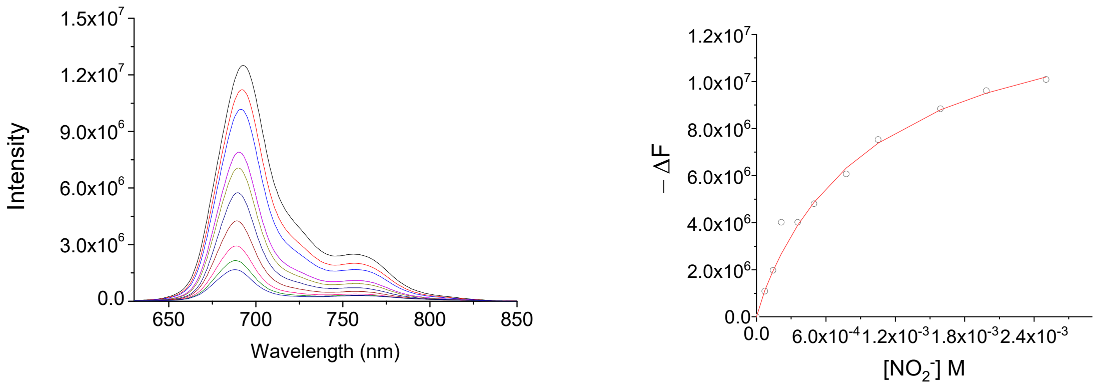

Figure S41. – Titration of **Pc1** with nitrite anion (left), and the plot of the experimental data and corresponding fit to a 1:2 binding model (right).

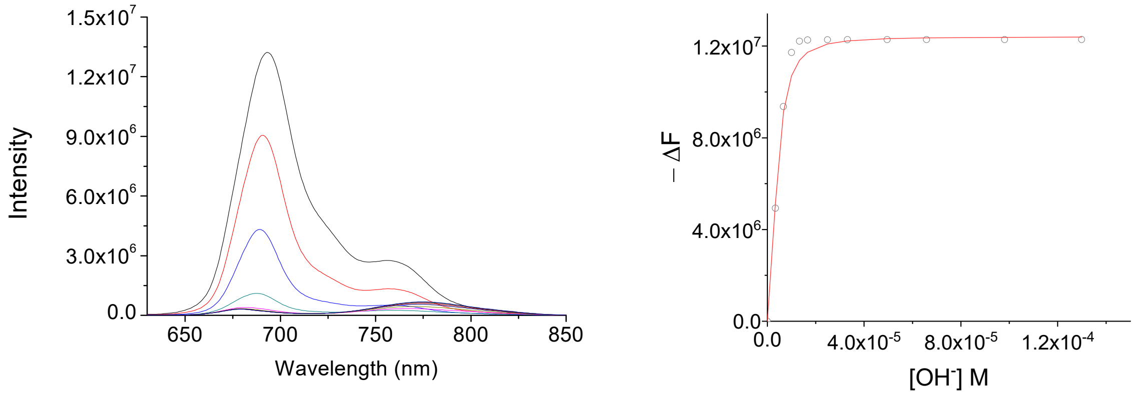

Figure S42. Titration of **Pc1** with hydroxide anion (left), and the plot of the experimental data and corresponding fit to a 1:2 binding model (right).

3.2. Titration of MSNP-Pc1 with anions (tetrabutylammonium salts) in DMSO

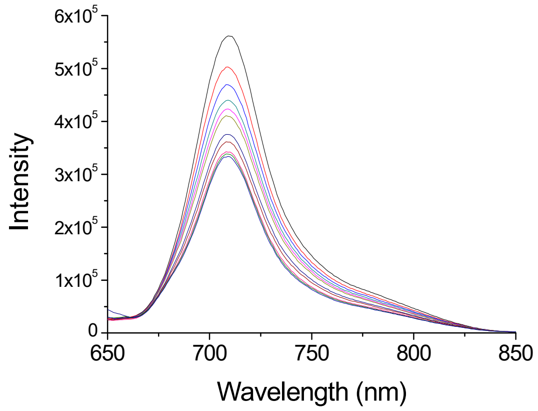

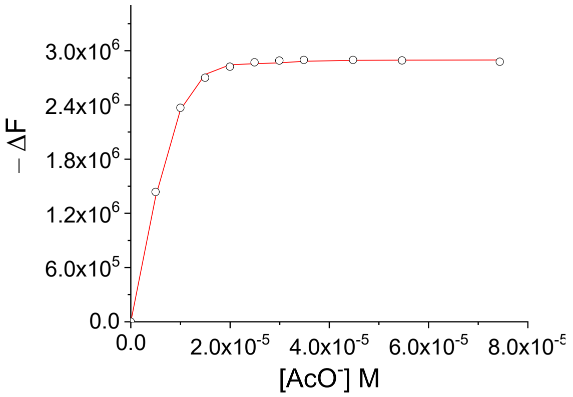

Figure S43. Titration of **MSNP-Pc1** with acetate anion (left), and the plot of the experimental data and corresponding fit to a 1:2 binding model (right).

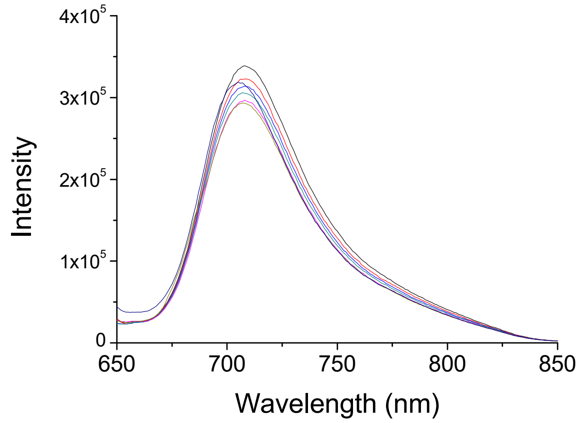

Figure S44. Titration of **MSNP-Pc1** with bromide anion.

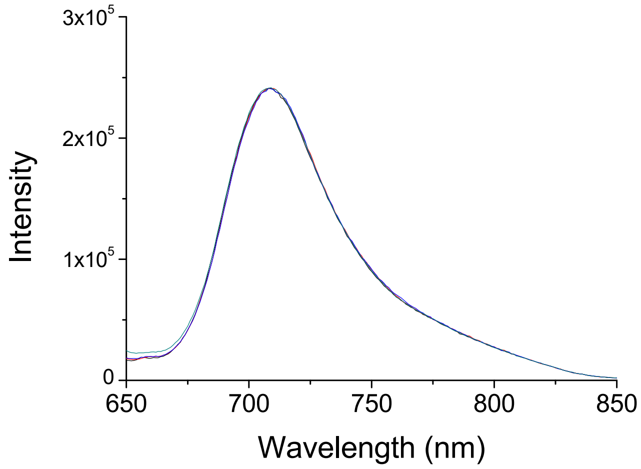

Figure S45. Titration of **MSNP-Pc1** with chloride anion.

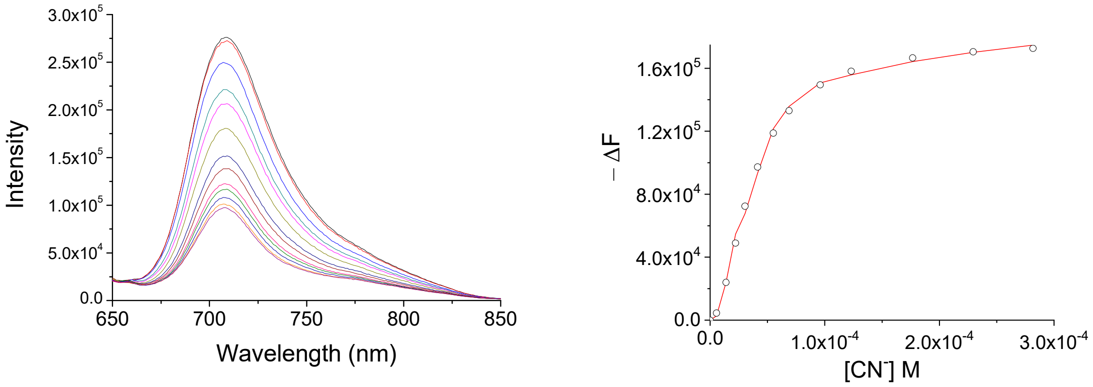

Figure S46. Titration of **MSNP-Pc1** with cyanide anion (left), and the plot of the experimental data and corresponding fit to a 1:2 binding model (right).

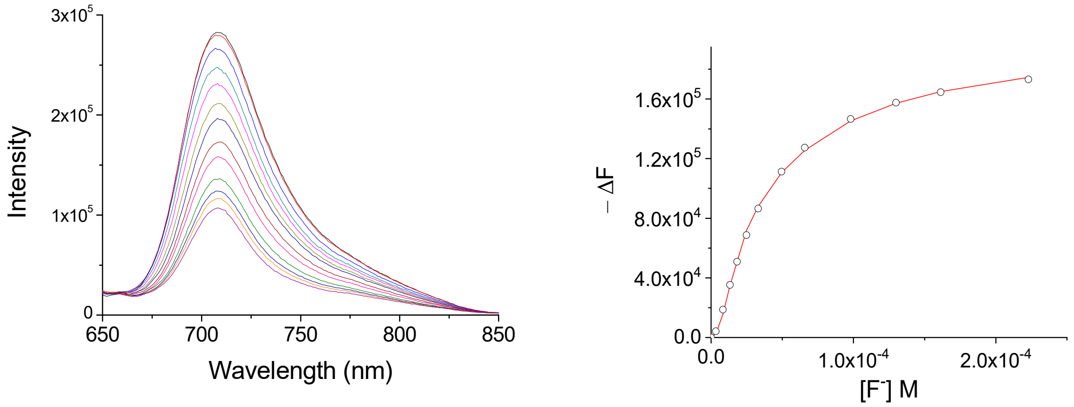

Figure S47. Titration of **MSNP-Pc1** with fluoride anion (left), and the plot of the experimental data and corresponding fit to a 1:2 binding model (right).

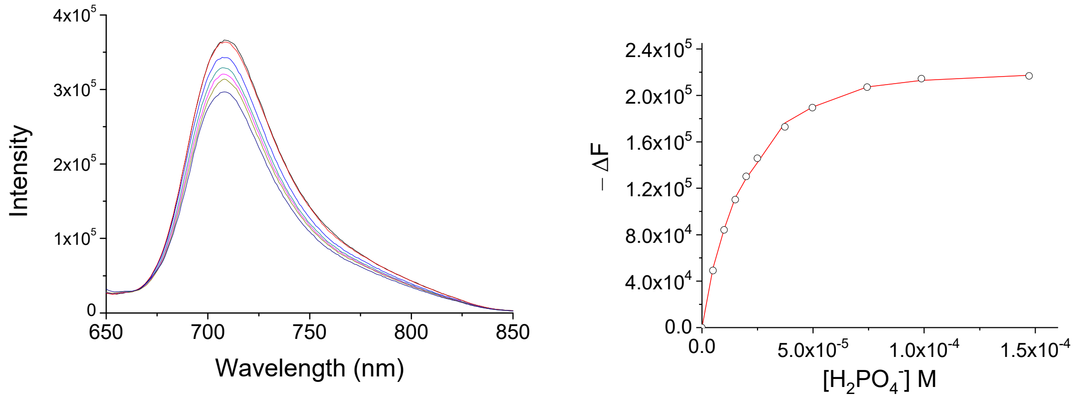

Figure S48. Titration of **MSNP-Pc1** with dihydrogen phosphate anion (left), and the plot of the experimental data and corresponding fit to a 1:2 binding model (right).

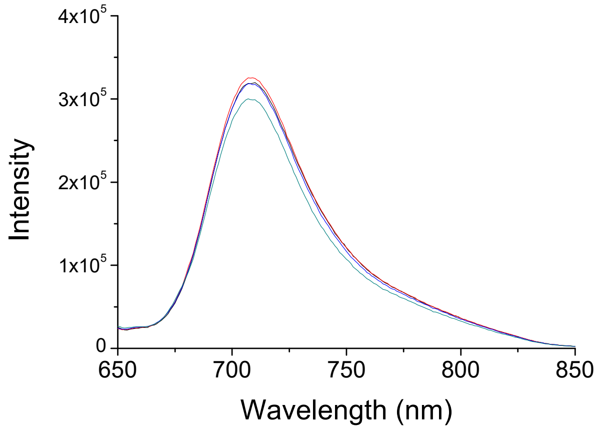

Figure S49. Titration of **MSNP-Pc1** with hydrogen sulfate anion.

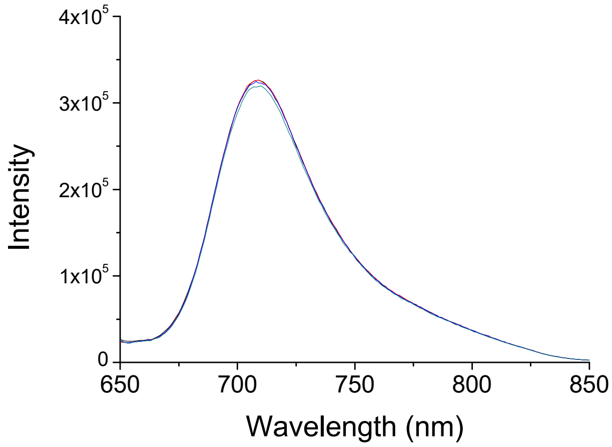

Figure S50. Titration of **MSNP-Pc1** with nitrate anion.

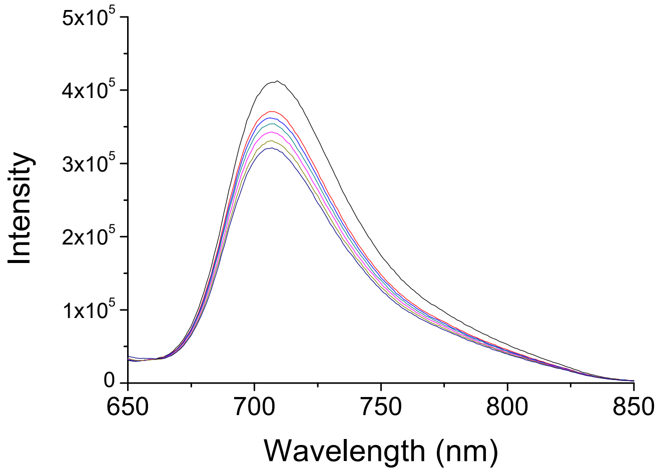

Figure S51. Titration of **MSNP-Pc1** with nitrite anion.

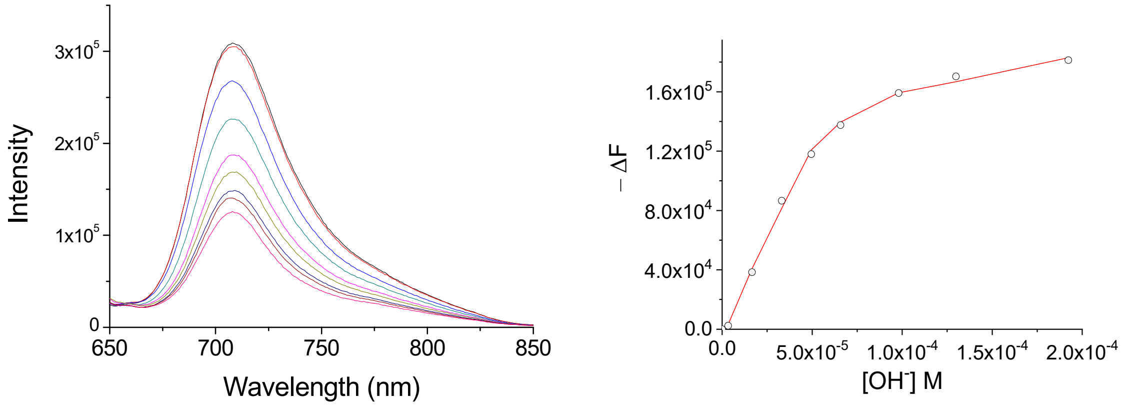

Figure S52. Titration of **MSNP-Pc1** with hydroxide anion (left), and the plot of the experimental data and corresponding fit to a 1:2 binding model (right).

*3.3. Titration of **MSNP-Pc1** with anions (tetrabutylammonium salts) in water*

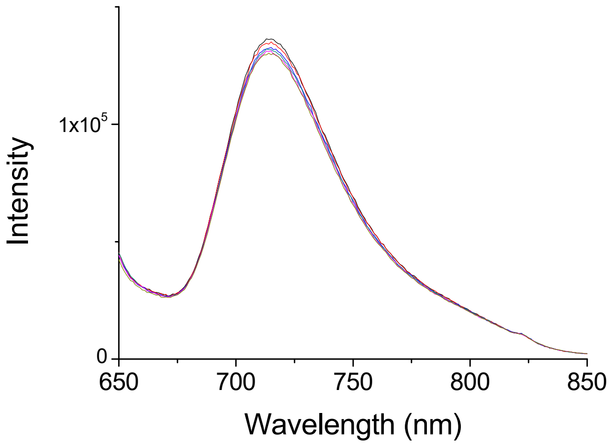

Figure S53. Titration of **MSNP-Pc1** with acetate anion.

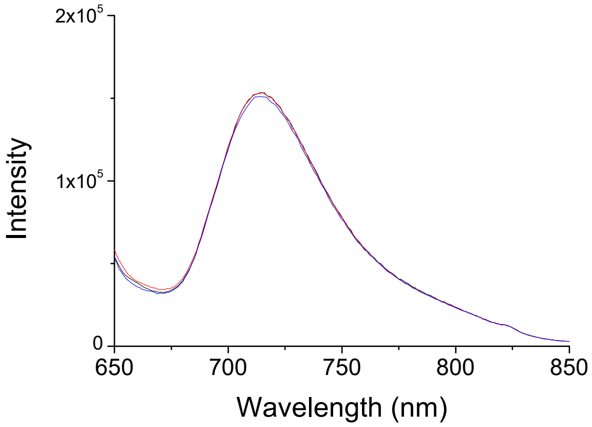

Figure S54. Titration of **MSNP-Pc1** with bromide anion.

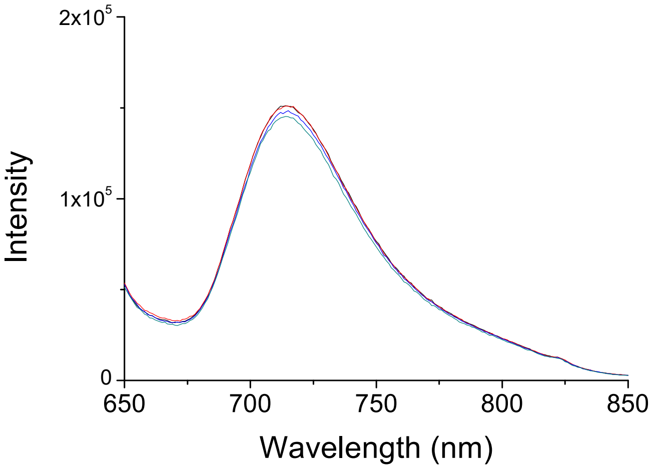

Figure S55. Titration of **MSNP-Pc1** with chloride anion.

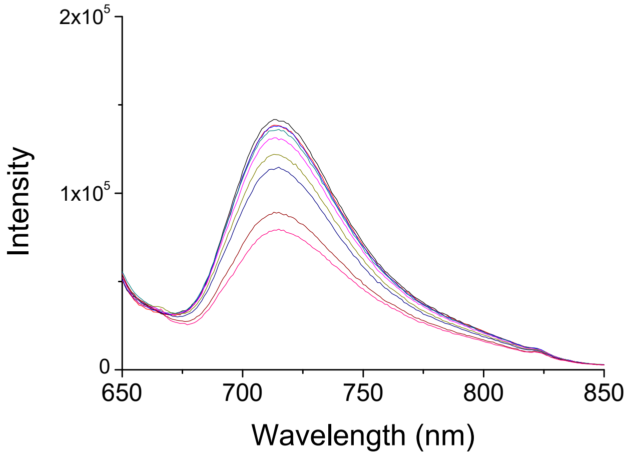

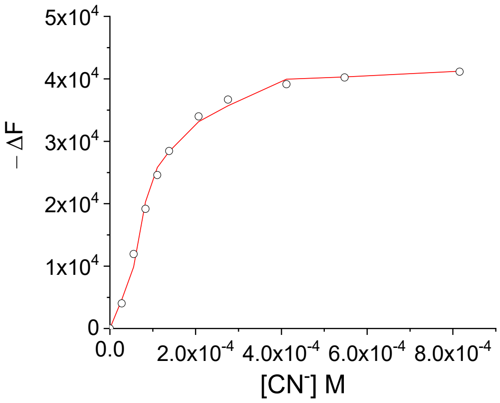

Figure S56. Titration of **MSNP-Pc1** with cyanide anion (left), and the plot of the experimental data and corresponding fit to a 1:2 binding model (right).

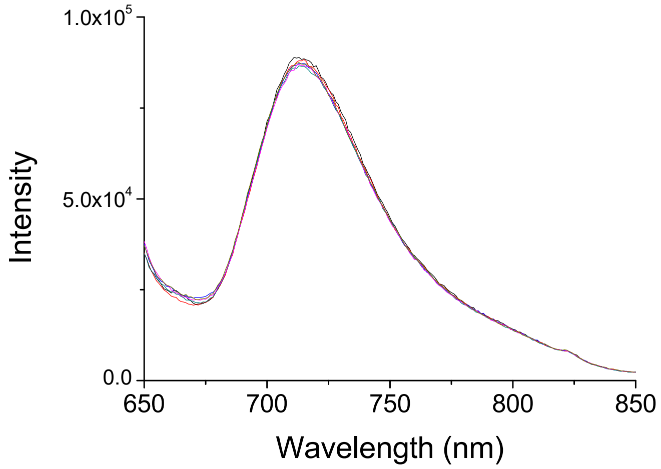

Figure S57. Titration of **MSNP-Pc1** with fluoride anion.

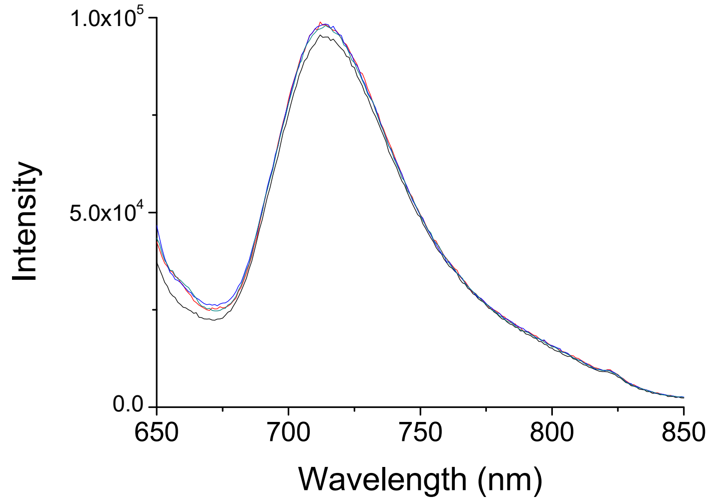

Figure S58. Titration of **MSNP-Pc1** with dihydrogen phosphate anion.

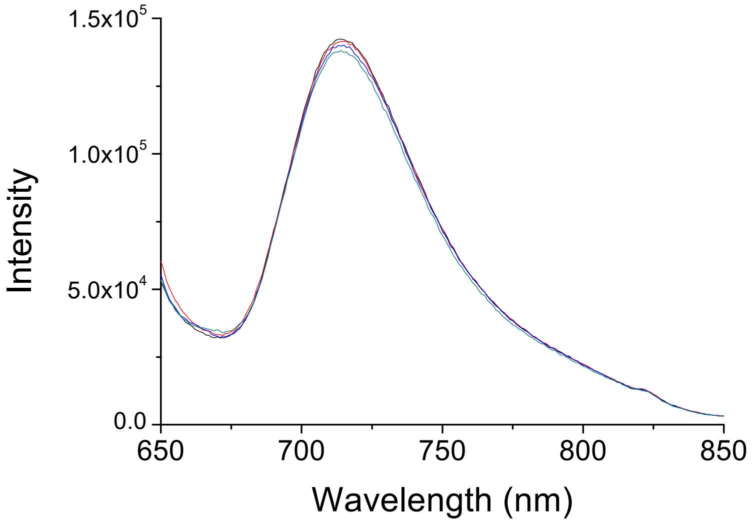

Figure S59. Titration of **MSNP-Pc1** with hydrogen sulfate anion.

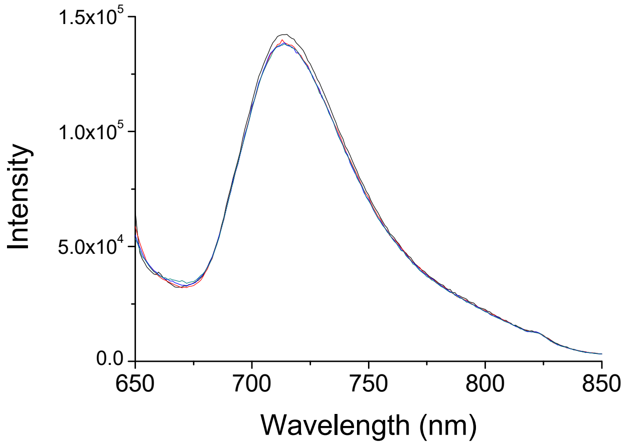

Figure S60. Titration of **MSNP-Pc1** with nitrate anion.

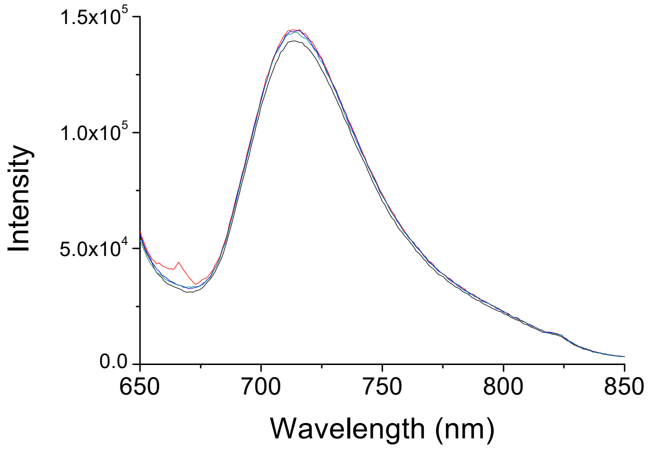

Figure S61. Titration of **MSNP-Pc1** with nitrite anion.
